# Unmasking genetic etiologies in neurodevelopmental disorders characterized by Cerebral Palsy: insights from integrative genomic approaches

**DOI:** 10.3389/fneur.2026.1742186

**Published:** 2026-02-23

**Authors:** Ayca Yigit, Ozlem Akgun-Dogan, Zeynep Ozkeserli, Gunseli Bayram Akcapınar, Semih Ayta, Pinar Gencpinar, Hulya Maras Genc, Busra Kutlubay, Bulent Kara, Hatice Gulhan Sozen, Nihat Bugra Agaoglu, Ozkan Ozdemir, Kaya Bilguvar, Ugur Ozbek

**Affiliations:** 1 Genome Studies, Graduate School of Health Sciences, Acibadem Mehmet Ali Aydinlar University, Istanbul, Türkiye; 2 Izmir International Biomedicine and Genome Institute, Dokuz Eylul University, Izmir, Türkiye; 3 Rare and Undiagnosed Disease Platform, IBG – Izmir Biomedicine and Genome Center, Izmir, Türkiye; 4 Rare Diseases and Orphan Drugs Application and Research Center (ACURARE), Acibadem Mehmet Ali Aydinlar University, Istanbul, Türkiye; 5 Department of Medical Genetics, School of Medicine, Acibadem Mehmet Ali Aydinlar University, Istanbul, Türkiye; 6 Department of Child Health and Diseases, School of Medicine, Acibadem Mehmet Ali Aydinlar University, Istanbul, Türkiye; 7 Department of Translational Medicine, Graduate School of Health Sciences, Acibadem Mehmet Ali Aydinlar University, Istanbul, Türkiye; 8 Department of Medical Biotechnology, Graduate School of Health Sciences, Acibadem Mehmet Ali Aydinlar University, Istanbul, Türkiye; 9 Spastic Children’s Foundation of Turkey, Istanbul, Türkiye; 10 Department of Pediatric Neurology, School of Medicine, Katip Celebi University, Izmir, Türkiye; 11 Pediatric Neurology, Umraniye Training and Research Hospital, University of Health Sciences, İstanbul, Türkiye; 12 Department of Pediatric Neurology, School of Medicine, Kocaeli University, Kocaeli, Türkiye; 13 Department of Pediatric Neurology, School of Medicine, Bahcesehir University, Istanbul, Türkiye; 14 Department of Medical Genetics, Division of Cancer Genetics, Umraniye Training and Research Hospital, Istanbul, Türkiye; 15 Department of Neurology, Krankenhaus Nordwest, Frankfurt, Germany; 16 Department of Medical Biology, School of Medicine, Acibadem Mehmet Ali Aydinlar University, Istanbul, Türkiye; 17 Departments of Neurosurgery and Genetics, Yale Center for Genome Analysis, Yale School of Medicine, New Haven, CT, United States

**Keywords:** Cerebral Palsy, CP genetics, genomic, neurodevelopmental disorders, next-generation sequencing

## Abstract

**Introduction:**

Cerebral Palsy (CP) is characterized by permanent, non-degenerative motor function deficits with increasing evidence of genetic contributions. Although prenatal and perinatal risk factors are well recognized, the underlying etiopathology remains incompletely understood. This study aimed to improve diagnostic accuracy and elucidate the genetic architecture of CP and CP-like phenotypes through systematic genomic analyses.

**Methods:**

Patients with clinically confirmed CP or CP-like presentations were recruited, and biological samples were stored in the ACU-Biobank. Whole-exome and whole-genome sequencing data were analyzed using a validated in-house pipeline incorporating comprehensive variant filtering, prioritization, and re-phenotyping.

**Results:**

Pathogenic or likely pathogenic variants were identified in 36.4% (24/66) of patients, while variants of uncertain significance (VUS) were detected in 25.8% (17/66). Identified variants involved genes such as *SPAST*, *KIF1A*, *PLA2G6*, *CTNNB1*, *L1CAM*, and *SYNGAP1*. These results demonstrate a substantial contribution of rare monogenic variants to CP and CP-like phenotypes, reflecting extensive genetic heterogeneity.

**Discussion:**

Our findings support the increasing evidence that genetic factors contribute significantly to CP etiology and emphasize the importance of integrating genomic testing into clinical evaluation. The systematic use of exome and genome sequencing improves diagnostic yield and enables genotype-informed classification, aiding targeted management and genetic counseling for affected individuals.

## Introduction

Cerebral Palsy (CP) refers to a broad and diverse group of non-progressive motor disorders characterized by difficulties in movement and posture. Rather than being a single, well-defined condition, CP is a clinical umbrella term for various disorders that share similar symptoms but can stem from different underlying causes (1, 2). These disorders arise from disruptions in brain development during the fetal period or within the first 2 years of life (3). They are often accompanied by comorbidities, such as intellectual disability (ID), epilepsy, visual and/or hearing impairments, and autism spectrum disorder (ASD) (4, 5).

CP is a leading cause of motor disabilities in children, with an estimated prevalence of 1.6 per 1,000 live births in high-income countries (HICs) (6). However, in low- and middle-income countries (LMICs), the numbers are noticeably higher, with rates reaching up to 3.4 per 1,000 live births. This difference highlights the challenges posed by disparities in healthcare access, the quality of perinatal care, and the broader social and economic conditions that shape maternal and infant health outcomes (6).

CP has been associated with a wide range of environmental risk factors occurring during the prenatal, perinatal, and postnatal periods. Nevertheless, the precise pathogenic mechanisms remain incompletely understood, and no single factor has been conclusively identified as a definitive cause (7). In general, the etiology of CP is multifactorial, encompassing environmental, genetic, and epigenetic contributors. Environmental risks include multiple gestations, prematurity, placental abnormalities, intrauterine growth restriction, and perinatal complications such as hypoxic–ischemic injury or infections (8, 9).

In recent years, advances in genomic technologies have highlighted the significant contribution of genetic factors to CP (10). Current research suggests that genetic variants may account for at least one-third of all CP cases (11, 12). The genetic architecture of CP is notably heterogeneous, with new genes continuously being discovered and linked to the condition. This expanding knowledge base emphasizes the critical importance of periodic reanalysis of genomic data. Recently, several studies have increasingly utilized family-based exome sequencing (ES) and genome sequencing (GS) as first-line diagnostic tools to investigate the complex genomic landscape of CP (10, 13). Compared with conventional approaches such as targeted gene panels or chromosomal microarray analysis (CMA), ES and GS provide a more comprehensive genetic assessment, resulting in substantially improved diagnostic yields (14–16). Due to their cost-effectiveness and robust performance, targeted gene panels and ES are currently preferred over GS in many clinical settings. Importantly, ES can generate data on genes not yet associated with disease, which increases the diagnostic potential over time through reanalysis. Indeed, reanalysis of ES data has been shown to uncover previously undiagnosed cases, with studies reporting diagnostic yield increases of 10–25% in patients where initial analyses failed to identify a genetic cause (17–20).

In this study, we aimed to elucidate the molecular etiology of CP cases without identified cause in a Turkish cohort by combining re-phenotyping and reanalysis of genomic data with advanced next-generation sequencing (NGS) approaches, including clinical exome sequencing and short-read whole-genome sequencing. State-of-the-art tools for variant interpretation and prioritization were employed to maximize diagnostic yield.

## Materials and methods

### Ethics statement

This study was approved by the Acıbadem Mehmet Ali Aydinlar University, and Acıbadem Healthcare Institutions Medical Research Ethics Committee (ATADEK) under decision number 2022–04/18, Spastic Children’s Foundation of Turkey (aka Cerebral Palsy Turkey) academic board, with permission number TSCV.200.023.080 in compliance with the principles of the Declaration of Helsinki (21). Informed consent was obtained from all participants or their legal guardians prior to their inclusion in the study.

### Study cohort

Patients with a preliminary clinical diagnosis of CP were recruited from the Turkey Spastic Children Foundation Clinics, Umraniye Research and Training Hospital Pediatric Genetics and Pediatric Neurology Units, and the Acıbadem Mehmet Ali Aydinlar University, ACURARE Undiagnosed Disease Program—Patient Experience Center between 2018 and 2021. Medical records of patients were retrospectively reviewed.

The study cohort was defined to reduce heterogeneity and potential confounding factors. Patients with a well-defined non-genetic etiology of Cerebral Palsy (CP) were excluded, including those with documented brain injuries such as severe hypoxic–ischemic events (e.g., cord rupture), intraventricular hemorrhage from extreme prematurity, or other acquired perinatal insults. Additionally, individuals with very premature birth (<30 weeks of gestation), severe perinatal asphyxia, known causes like clotting disorders, severe perinatal infections, central nervous system tumors, trauma, or previously confirmed genetic diagnoses were excluded. Patients presenting with prenatal, perinatal, or postnatal risk factors were included only when no definitive non-genetic etiology could be established, as the presence of such risk factors does not preclude an underlying genetic cause. The inclusion criteria consisted of patients aged 0–18 years with a preliminary clinical diagnosis of CP who had undergone clinical exome sequencing, whole-exome sequencing, or whole-genome sequencing without identifying a genetic etiology.

A total of 66 unsolved CP cases were included in the study cohort. Raw data files in Fast Adaptive Shrinkage Thresholding Algorithm and Quality format (FASTQ) obtained from previous clinical exome sequencing (CES), trio whole exome sequencing (WES) and trio whole genome sequencing (WGS) experiments were subsequently obtained. All patients’ medical records, and when needed, in-person assessments of the patients, were assessed by a single pediatric geneticist for deep phenotyping, with the resulting HPO terms utilized in the reanalysis process. Collected data were stored in the Acıbadem Mehmet Ali Aydinlar University, Biobank Unit alongside clinical and demographic information with the informed consent of the patients/ families in accordance with strict patient privacy procedures of the Biobank.

### Genome analysis

FASTQ files of CES, WES and WGS data were processed with an in-house genome analysis pipeline, GENNEXT,^1^ platform (22). Sequence reads were aligned to the reference genome (GRCh38) independently at each site using SNAP Aligner (23), followed by post-alignment processing with elPrep (24), encompassing duplicate marking, indel realignment, and recalibration of base quality scores. Single-nucleotide variants (SNV) and small indels were called using GATK HaplotypeCaller (25), and DeepVariant Caller (26), annotated using the GENNEXT annotation tool.

### Variant filtering and prioritization

We prioritized variants that met the following criteria: (1) rarity, with a minor allele frequency of 0.0005 (0.005 for recessive inheritance) in population databases such as gnomAD (27, 28) and Turkish Variome (29); (2) variants occurring within coding regions and canonical splice sites; including missense, nonsense, frameshift, start-loss, stop-loss, and canonical splice-site variants, as well as variants predicted to alter splicing; (3) prediction as truncating (including frameshift, stop-gains, and stop-losses), canonical splice sites, missense, or inframe indels by multiple in silico prediction tools, including CADD (30), Polyphen-2 (31), MetaSVM (32), SIFT (33), and SpliceAI (34); (4) integration of curated clinical databases, including OMIM and ClinVar, to assess known gene–disease associations, as identified through comprehensive literature and Genomic England PanelApp;^2^ and (5) final variant classification performed according to the American College of Medical Genetics and Genomics and the Association for Molecular Pathology (ACMG/AMP) (35) guidelines, integrating population data, computational evidence, functional consequence, and segregation information when available. The stepwise prioritization workflow is summarized in Supplementary Figure 1.

### Functional enrichment and protein network analysis

To evaluate the functional coherence of the genes prioritized through variant interpretation in the cohort, protein–protein interaction (PPI) network construction and functional enrichment analyses were performed using the STRING web tool (v12.0).^3^ The analyses were conducted using genes harboring pathogenic/likely pathogenic (P/LP) variants together with clinically relevant variants of uncertain significance (VUS), in order to capture shared functional relationships across the full set of candidate disease-associated genes. PPI enrichment *p*-values were calculated using a hypergeometric test, comparing the observed number of interactions to that expected by chance for a gene set of similar size.

For functional enrichment, StringDB identifies over-represented Gene Ontology (GO) terms, KEGG pathways,^4^ and disease associations, applying Benjamini–Hochberg correction to control the false discovery rate (FDR). The same gene set (P/LP + clinically relevant VUS) was used for GO enrichment analysis. Terms with corrected FDR < 0.05 were considered significantly enriched. In addition, enrichment strength scores, defined as the log₁₀ ratio of observed to expected gene counts, were used to quantify effect size, and terms with a strength greater than 0.5 were reported to highlight biologically meaningful associations. All statistical analyses were performed as implemented in the STRING web platform, using its default settings unless otherwise specified.

## Results

### CP cohort characteristics

**Figure 1:**
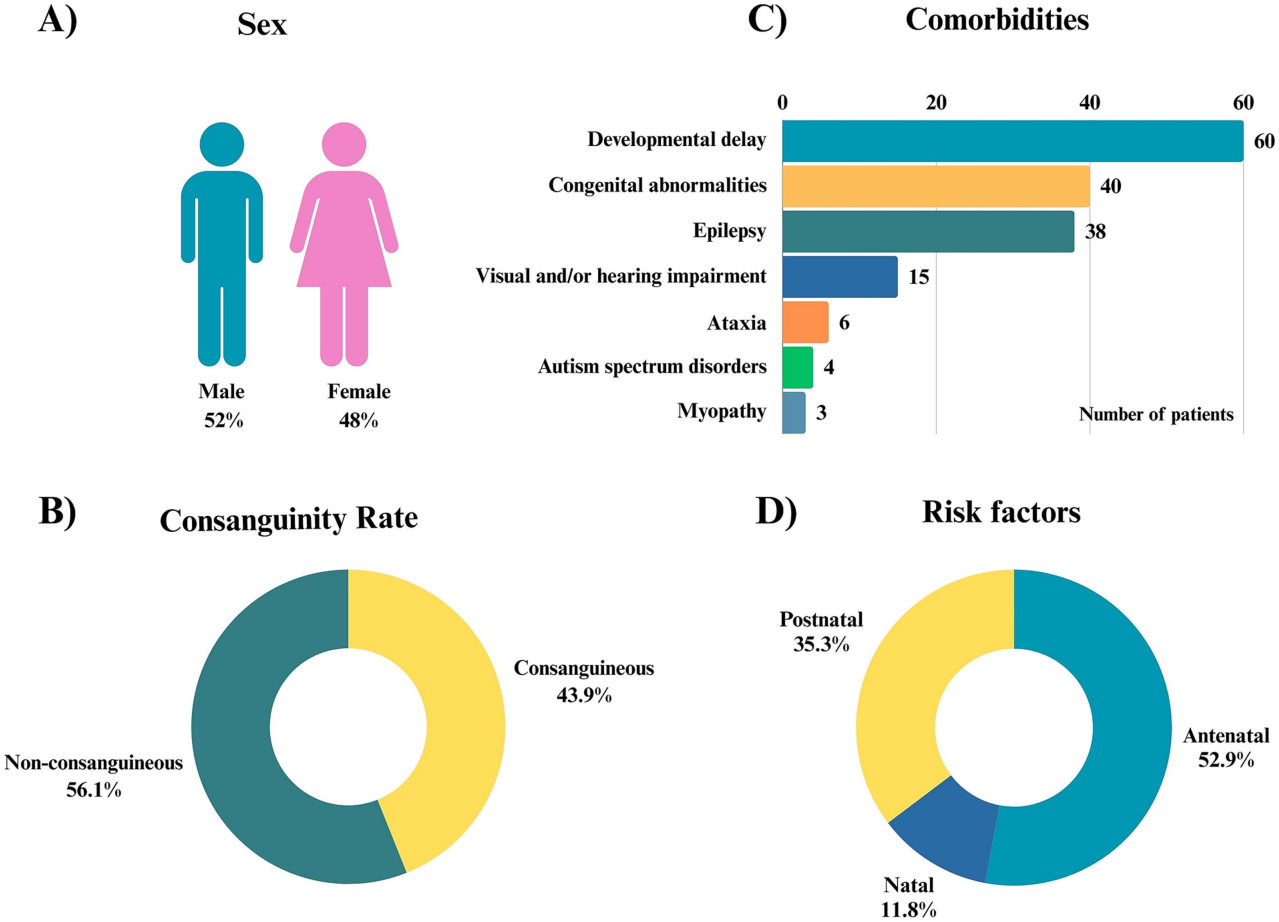
Clinical and demographic characteristics of the cohort. **(A)** Distribution of sex among the patients (male: 52%, female: 48%). **(B)** Parental consanguinity rate, with 43.9% of patients born to consanguineous parents and 56.1% to non-consanguineous parents. **(C)** Frequency of comorbidities observed in the cohort. The most common comorbidity was developmental delay (*n* = 60), followed by congenital abnormalities (*n* = 40), epilepsy (*n* = 38), and visual and/or hearing impairment (*n* = 15). Less common comorbidities included ataxia (*n* = 6), autism spectrum disorders (*n* = 4), and myopathy (*n* = 3). **(D)** Identified risk factors (*n* = 34) were classified according to the timing of occurrence as antenatal (52.9%), natal (11.8%), or postnatal (35.3%). Classification was based on whether the risk factor occurred before delivery (antenatal), during labor and delivery (natal), or after birth (postnatal). Percentages represent event-based frequencies, as more than one risk factor could be present in the same patient.

A total of 66 patients were included in the study. The cohort comprised 32 females and 34 males (Figure 1A). The mean age of the cohort was 6.0 years, ranging from 3 months to 18 years. Consanguinity was identified in approximately 44% (29/66) of families (Figure 1B).

All patients presented with at least one comorbidity. Developmental delay was the most frequently reported comorbidity, observed in more than half of the cohort, followed by congenital anomalies and epilepsy. The reported comorbidities and their distribution are illustrated in Figure 1C. Detailed clinical findings and corresponding HPO terms for each individual are provided in Table 1; Supplementary Table 1 summarizes the demographic and clinical characteristics of the cohort.

**Table 1:** Clinical details of Turkish CP cohort.

Patient ID	Sex	Age at last examination	Cons.	Phenotypic features
CP_P1.1	F	5,0	Yes	Facial dysmorphism, global developmental delay, ataxia, cerebellar vermis hypoplasia, periventricular white matter hyperintensities and ectopic posterior pituitary
CP_P2.1	M	9,0	No	Gastroesophageal reflux, global developmental delay, seizures, dysphagia, hypoplasia of the corpus callosum, ventriculomegaly, frontotemporal cerebral atrophy, and calcification of the globus pallidus
CP_P3.1	F	11,0	NA	Scoliosis, global developmental delay, hypotonia, and intellectual disability
CP_P4.1	M	11,2	Yes	Global developmental delay, motor delay and intellectual disability
CP_P5.1	F	4,1	No	Global developmental delay, hypotonia, ataxia, cerebral atrophy, and drooling
CP_P6.1	F	0,6	NA	Facial dysmorphism, nuchal translucency, biparietal narrowing, and neonatal hypotonia
CP_P7.1	F	9,0	Yes	Facial dysmorphism, lower limb spasticity, myopathy, muscular dystrophy, distal prominence of the metatarsals, global developmental delay, hypotonia, seizures, dysphagia, dysplasia of the corpus callosum, and elevated serum creatine phosphokinase
CP_P8.1	M	0,6	NA	Distal arthrogryposis
CP_P9.1	M	9,0	NA	Facial dysmorphism, neonatal asphyxia, scoliosis, spastic diplegia, pes planus, cryptorchidism, hyperreflexia and feeding difficulties
CP_P10.1	F	0,3	Yes	The high palate, neonatal respiratory distress, talipes equinovarus, facial hypotonia, polyneuropathy, and periventricular white matter hyperintensities
CP_P11.1	M	12,0	NA	Microcephaly, and intellectual disability
CP_P12.1	M	2,0	NA	Coarse facial features, global developmental delay, seizures, widespread papulopustular rash on the anterior and posterior surfaces of the chest
CP_P13.1	F	7,3	Yes	Microcephaly, global developmental delay, motor delay, seizures, spasticity, absent speech, bruxism, hyperacusis, dysmorphic facial features, difficulty walking, limited knee, elbow flexion, and periventricular white matter hyperintensities
CP_P14.1	F	1,2	Yes	Short neck, hypertelorism, scoliosis, global developmental delay, hypotonia, seizures and spasticity
CP_P15.1	M	2,9	No	Global developmental delay, absent speech, seizures, spasticity, hydrocephalus, intellectual disability, motor delay, cerebral atrophy, bruxism, and hypoplasia of the corpus callosum
CP_P16.1	M	6,0	No	Facial dysmorphism, retrognathia, hypertelorism, talipes equinovarus, pes cavus, hypospadias, cryptorchidism, global developmental delay, delayed speech and language development, and agenesis of the corpus callosum
CP_P17.1	F	12,8	No	Bilateral hearing impairment, poor head control, global developmental delay, delayed speech and language development, learning difficulties, motor delay, increased deep tendon reflexes, inguinal hernia, thrombocytopenia, proteinuria and hematuria
CP_P18.1	M	5,3	Yes	Strabismus, global developmental delay, seizures, motor delay, neurodevelopmental delay, deficiency in independent sitting, chronic infection and continuous fever
CP_P19.1	F	8,8	Yes	Microcephaly, intellectual disability, long palpebral fissures, macrodontia, global developmental delay, delayed speech and language development, and seizures
CP_P20.1	F	2,4	No	Pes planus, global developmental delay, motor delay, epileptic encephalopathy (under follow-up with a diagnosis of CP)
CP_P21.1	M	5,6	No	Retinal detachment, and hypotonia
CP_P22.1	F	7,1	Yes	Microcephaly, hypertonia, nephrotic syndrome, global developmental delay, seizures, spasticity, and elevated serum creatine phosphokinase
CP_P23.1	F	1,1	No	Global developmental delay, hypotonia, seizures and muscle spasms
CP_P24.1	M	1,7	Yes	Microcephaly, facial dysmorphism, neonatal hypotonia, hypertonia, seizures, agenesis of the corpus callosum, hyperreflexia, dilation of lateral ventricles and neurodevelopmental delay
CP_P25.1	F	NA	Yes	Microcephaly, pectus excavatum, seizures, motor delay and frontal cortical atrophy
CP_P26.1	F	0,4	No	Retrognathia, respiratory distress, flexion contracture, limited knee extension, wide intermammillary distance, arthrogryposis multiplex congenita, and poor suck
CP_P27.1	M	8,0	No	Retinitis pigmentosa inversa, hearing impairment, respiratory distress, arthritis, global developmental delay, delayed speech and language development, seizures, and ataxic gait
CP_P28.1	M	11,8	Yes	Global developmental delay, autism, absent speech, seizures, motor delay, polyneuropathy, and neurodevelopmental delay
CP_P29.1	M	0,5	Yes	Microcephaly, facial dysmorphism, visual impairment, tapered finger, global developmental delay, seizures, dystonia, motor delay, EEG abnormalities, diffuse cerebral atrophy, striatal T2 hyperintensity, dystonic gait, epileptic encephalopathy, and congenital hypothyroidism
CP_P30.1	F	5,4	Yes	Poor suck, scoliosis, torticollis, neonatal hypotonia, kyphosis, hip dislocation, myopathy, muscular dystrophy, and joint hypermobility
CP_P31.1	F	10,7	Yes	High narrow palate, strabismus, hypertelorism, difficulty walking, clinodactyly, global developmental delay, delayed speech and language development
CP_P32.1	F	8,7	Yes	Microcephaly, global developmental delay, neurodevelopmental delay
CP_P32.2	M	3,6	Yes	Microcephaly, global developmental delay, neurodevelopmental delay
CP_P33.1	M	0,3	Yes	Neonatal asphyxia, hypotonia and dysphagia
CP_P34.1	M	13,2	Yes	Facial asymmetry, seizures, homocystinuria, abnormalities of glutamine and homocysteine metabolism
CP_P35.1	M	9,0	No	Microcephaly, tracheomalacia, bronchomalacia, spastic tetraplegia, cryptorchidism, seizures, cerebellar atrophy, infantile spasms, and feeding difficulties
CP_P36.1	F	7,7	No	Nystagmus, strabismus, difficulty walking, global developmental delay, delayed speech and language development, seizures and agenesis of the corpus callosum
CP_P37.1	F	1,8	No	Retrognathia, facial dysmorphism, visual impairment, abnormal electroretinogram, neonatal hypotonia, seizures, motor delay, cerebral atrophy, increased deep tendon reflexes, global developmental delay, milk allergy and feeding difficulties
CP_P38.1	F	7,0	Yes	Microcephaly, intellectual disability, global developmental delay, seizures and autism
CP_P39.1	M	7,0	No	Facial dysmorphism, strabismus, prominent fingertip pads, global developmental delay, motor delay, polyhydramnios and prematurity
CP_P40.1	M	1,7	Yes	Brachycephaly, atrial septal defect, ventricular septal defect, autism, hypotonia, motor delay, self-injurious behavior, and inappropriate laughter
CP_P41.1	F	1,9	Yes	Global developmental delay, hypotonia, seizures, cerebellar vermis atrophy, dilation of lateral ventricles, aggressive behavior, inferior cerebellar vermis hypoplasia, abnormality of glycolysis and abnormality of creatine metabolism
CP_P42.1	F	2,9	No	Global developmental delay, delayed speech and language development, seizures, and EEG abnormality
CP_P43.1	M	5,1	No	Neonatal sepsis, microcephaly, seizures, delayed speech and language development, global developmental delay
CP_P44.1	M	4,9	No	Cerebral Palsy
CP_P45.1	M	3,1	No	Growth retardation, strabismus, scoliosis, pes cavus, abnormality of toe proximal phalanx, global developmental delay, spasticity and cerebellar vermis atrophy
CP_P46.1	M	8,4	Yes	Hypertonia, lower limb spasticity, global developmental delay, stereotypes, and EEG abnormality
CP_P47.1	F	1,8	No	Macrocephaly, facial dysmorphism, hypotonia, hydrocephalus, convulsive status epilepticus, dilation of lateral ventricles, and periventricular leukomalacia
CP_P48.1	M	10,7	No	Mild global developmental delay, seizures, cerebellar atrophy, agenesis of corpus callosum, reduced tendon reflexes, neonatal hypotonia, poor suck, cerebral atrophy, gait ataxia, hypoplasia of the corpus callosum, febrile seizures, dilation of lateral ventricles
CP_P49.1	M	2,9	No	Mild microcephaly, impaired mastication, laryngomalacia, pes planus, infantile axial hypotonia, delayed speech and language development, motor delay, reduced tendon reflexes, delayed myelination, and hyperintensity of cerebral white matter on MRI
CP_P50.1	M	7,8	Yes	Hypodontia, strabismus, nystagmus, difficulty walking, global developmental delay, seizures, motor delay and EEG abnormality
CP_P51.1	M	2,8	No	Delayed gross motor development, increased nuchal translucency, delayed speech and language development, nystagmus, astigmatism, multiple tooth decay, relative macrocephaly, polyneuropathy, hypotonia and pectus carinatum
CP_P52.1	F	9,0	No	Global developmental delay, seizures, ataxia, severe motor delay, absent speech, Intellectual disability, pontocerebellar hypoplasia, facial dysmorphism, vomiting
CP_P53.1	F	3,0	No	Global developmental delay, poor suck, Cerebral Palsy, axial hypotonia, hypertonia, inability to walk, absent speech, stereotypical hand wringing
CP_P54.1	F	3,0	No	Microcephaly, hypertonia, poor head control, global developmental delay, cerebellar atrophy, hypoplasia of the corpus callosum, dilation of lateral ventricles, generalized hypotonia, seizures, dystonia
CP_P55.1	M	7,0	No	Global developmental delay, seizures, hand tremor, Inability to walk, absent speech, hydrocephalus
CP_P55.2	F	2,0	No	Global developmental delay, seizures, EEG abnormality
CP_P56.1	M	6,0	No	Motor delay, spasticity, delayed speech and language development, delayed gross motor development, difficulty walking, hyperactive deep tendon reflexes, broad hallux, pes cavus
CP_P57.1	F	11,0	No	Visual impairment, upslanted palpebral fissures, seizures, cerebellar atrophy, generalized hypotonia, lower limb spasticity, delayed gross motor development, difficulty walking, knee flexion contracture, cerebellar vermis atrophy, pes valgus
CP_P58.1	F	6,0	Yes	Neonatal respiratory distress, brachycephaly, retrognathia, narrow forehead, proptosis, absent speech, seizures, spasticity, hydrocephalus, partial agenesis of corpus callosum, EEG abnormality, inability to walk, colpocephaly
CP_P59.1	M	0,7	Yes	Global developmental delay, generalized hypotonia, round face, poor head control, spasticity
CP_P60.1	F	3,0	Yes	Facial dysmorphism, microcephaly, hypertonia, rocker bottom root, global developmental delay, motor delay, generalized tonic–clonic seizures, ventriculomegaly, delayed myelination, generalized hypotonia
CP_P61.1	M	9,0	No	Seizures, intellectual disability, spastic paraplegia, spasticity, global developmental delay, delayed ability to walk, difficulty walking,
CP_P62.1	M	18,0	Yes	Stereotypy, seizures, global developmental delay, spastic tetraparesis, tremor, absent speech, EEG abnormality, difficulty walking, hyperactive deep tendon reflexes, neurodevelopmental delay, delayed ability to walk
CP_P63.1	F	18,0	Yes	Global developmental delay, mild intellectual disability, delayed speech and language development, spasticity, difficulty walking, pes cavus, joint laxity
CP_P64.1	M	10,0	No	Global developmental delay, seizures, intellectual disability, motor regression, developmental regression, absent speech, inability to walk, EEG abnormality

Most patients had no documented history of risk factors. However, 37.9% (25 of 66) of the cohort had at least one identified risk factor associated with CP, including prematurity, small for gestational age, postnatal respiratory distress, polyhydramnios, maternal vaginal bleeding, breech delivery, neonatal sepsis, or intrauterine growth restriction (Figure 1D, summarizing prenatal, natal, and postnatal risk factor events identified in the cohort).

### Genome analysis

**Figure 2:**
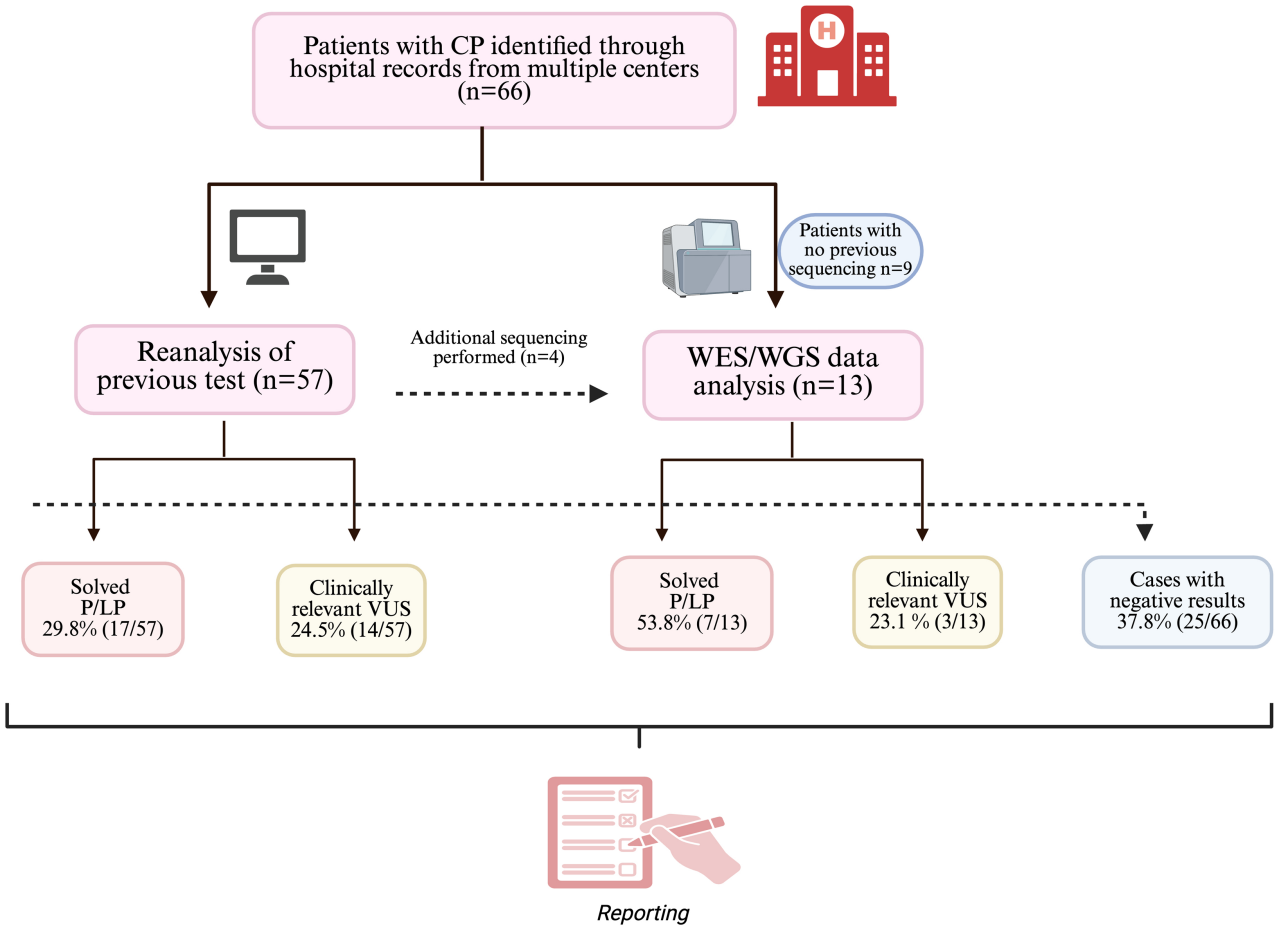
Overview of genomic analysis strategies and diagnostic outcomes in the study cohort. Genomic analyses were performed in a cohort of 66 patients with CP and CP-like phenotypes identified through hospital records from multiple centers. Two complementary approaches were applied: reanalysis of previously generated exome sequencing (CES or WES) data (*n* = 57) and analysis of newly generated trio-based WES or WGS data (*n* = 13). P/LP variants fulfilling ACMG/AMP criteria were considered to provide a definitive molecular diagnosis, while VUSs were reported separately as clinically relevant candidate findings. Cases without a definitive molecular diagnosis were classified as genetically unresolved.

Genomic analyses were performed in a cohort of 66 unsolved CP and CP-like cases using two complementary strategies. For 57 individuals, existing clinical exome sequencing (CES) or whole-exome sequencing (WES) data was conducted, depending on data availability and quality. In 13 cases, newly generated trio-based whole-exome sequencing (WES) or whole-genome sequencing (WGS) data were analyzed, including unaffected parents and, when available, affected or unaffected siblings (Figure 2).

Using these complementary approaches, a definitive molecular diagnosis based on the identification of pathogenic or likely pathogenic (P/LP) variants was established in 24 of 66 patients (36.4%). Table 2 summarizes the pathogenic and likely pathogenic variants found in individuals with a definitive genetic diagnosis, providing detailed annotations of variant types and their ACMG classifications.

**Table 2:** Pathogenic and likely pathogenic variants identified in the cohort and clinical and genetic information of patients harboring these variants.

Patient ID	Gene	Variant	GnomAD v4 allele Freq.	variant type	DISORDER (MIM)	CP risk factors	Zyg (segregation)	ACMG classification
CP_P17.1	*ASXL1* *SETBP1*	NM_015338.6: c.2077C > T (p. Arg693Ter) NM_015559.3: c.2612 T > C (p. Ile871Thr)	0.00001 6.196e-7	Stop gain Missense	Bohring-Opitz syndrome (MIM #605039) and Intellectual developmental disorder, autosomal dominant 29 (MIM #616078) Schinzel-Giedion midface retraction syndrome (MIM #269150)	Low birth weight	Het (NA) Het (NA)	P (PVS1, PS4, PM2, PP5) P (PM2, PS4, PM5, PM1, PP3, PP5)
CP_P39.1	*PPM1D*	NM_003620.4:c.1262C > A(p. Ser421Ter)	6.300e-7	Stop gain	jansen-de Vries syndrome (MIM #617450)	Polyhydramnios, vaginal bleeding, prematurity and low birth weight	Het (NA)	P (PVS1, PS4, PM2, PP5)
CP_P8.1	*COL6A1*	NM_001848.3: c.1693C > T (p. Arg565Ter)	0.000001	Stop gain	Ulrich congenital muscular dystrophy 1A (MIM #254090)	–	Hom (NA)	P (PVS1, PM2, PS4, PP5)
CP_P21.1	*CTNNB1*	NM_001904.4: c.1923dup (p. Glu642ArgfsTer6)	–	Frameshift	Exudative vitreoretinopathy 7 (MIM #617572) Neurodevelopmental disorder with spastic diplegia and visual defects (MIM #615075)	–	Het (NA)	P (PVS1, PM2, PS4, PP5)
CP_P30.1	*COL6A1*	NM_001848.3: c.811C > T (p. Arg271Ter)	0.000006	Stop gain	Ulrich congenital muscular dystrophy 1A (MIM #254090)	Breech presentation at birth	Hom (NA)	P (PVS1, PM2, PP5)
CP_P46.1	*CACNA1E*	NM_001205293.3:c.5365-3_5,365-2del	–	Splice site	Developmental and epileptic encephalopathy 69 (MIM #618285)	–	Het (*de novo*)	P (PVS1, PM2, PM6)
CP_P59.1	*NGLY1*	NM_018297.4:c.1533_1536del (p. Asn511Lysfs*51)	0.00001	Frameshift	Congenital disorder of deglycosylation 1 (MIM #615273)	–	Hom (maternal paternal)	P (PVS1, PM2, PM3, PP5)
CP_P61.1	*ATP1A3*	NM_152296.5:c.2324C > T (p. Pro775Leu)	–	Missense	Developmental and epileptic encephalopathy 99 (MIM #619606) CAPOS syndrome (MIM #601338)	–	Het (NA)	P (PS2, PM2, PM5, PM1, PP3, PP2, PP5)
CP_P14.1	*WWOX*	NM_016373.4: c.716 T > G (p. Leu239Arg)	–	Missense	Developmental and epileptic encephalopathy 28 (MIM #616211) Spinocerebellar ataxia, autosomal recessive 12 (MIM #614322)	Intrauterine growth retardation	Hom (maternal paternal)	P (PP3, PM3, PM2, PP5)
CP_P13.1	*SYNGAP1*	NM_006772.3: c.851 T > G (p. Leu284Arg)	–	missense	Intellectual developmental disorder, autosomal dominant 5 (MIM #612621)	Polyhydramnios and prolonged labor	Het (de novo)	P (PP3, PM2, PM5, PP2, PS2)
CP_P2.1	*PLA2G6*	NM_003560.4: c.1772G > A (p. Arg591Gln)	0.000008	Missense	Infantile neuroaxonal dystrophy (MIM #256600) Neurodegeneration with brain iron accumulation 2B (MIM #610217)	–	Hom (NA)	P (PM3, PM2, PM5, PM1, PP3, PP2, PP5)
CP_P62.1	*ST3GAL5*	NM_003896.4:c.601G > A (p. Gly201Arg)	0.000009	Missense	Salt and pepper developmental regression syndrome (MIM #609056)	–	Hom (maternal paternal)	P (PM2, PP3, PP5)
CP_P57.1	*KIF1A*	NM_001244008.2:c.946C > T (p. Arg316Trp)	–	Missense	NESCAV syndrome (MIM #614255) Spastic paraplegia 30, autosomal dominant (MIM #610357)	Low birth weight	Het (de novo)	P (PM2, PM5, PM1, PS2, PP3, PP2, PP5)
CP_P64.1	*STXBP1*	NM_001032221.6:c.578G > C (p. Gly193Ala)	–	Missense	Developmental and epileptic encephalopathy 4 (MIM #612164)	–	Het (de novo)	P (PM2, PM5, PM1, PP3, PP2, PP5, PS2)
CP_P12.1	*ARID1B*	NM_001374828.1:c.1391_1400del (p. Gly464fs)	–	Frameshift	Coffin-Siris syndrome 1 (MIM #135900)	Neonatal seizures	Het (NA)	LP (PVS1, PM2)
CP_P33.1	*COL1A2*	NM_000089.4:c.3275dup (p. Gly1093TrpfsTer17)	0.000001	Frameshift	Combined osteogenesis imperfecta and Ehlers-Danlos syndrome 2 (MIM #619120) Ehlers-Danlos syndrome, arthrochalasia type,2 (MIM #617821)	Neonatal respiratory distress	Het (NA)	LP (PVS1, PM2)
CP_P40.1	*LINS1*	NM_001040616.3:c.1870del (p. Gln624LysfsTer4)	–	Frameshift	Intellectual developmental disorder, autosomal recessive 27 (MIM #614340)	–	Hom (NA)	LP (PVS1, PM2)
CP_P31.1	*SAMD9*	NM_017654.4:c.2159del (p. Asn720ThrfsTer35)	0.000009	Frameshift	MIRAGE syndrome (MIM #617053)	Vaginal bleeding, and low birth weight	Het (NA)	LP (PVS1, PM2, PP5)
CP_P48.1	*L1CAM*	NM_001278116.2:c.414 > A (p. Trp138Ter)	–	Stop gain	Corpus callosum, partial agenesis of (MIM #304100) Hydrocephalus, congenital, X linked (MIM #307000) MASA syndrome (MIM #303350)	–	Hem (de novo)	LP (PVS1, PM2, PM6)
CP_P47.1	*PTEN*	NM_000314.8:c.275A > T (p. Asp92Val)	–	Missense	Cowden syndrome 1 (MIM #158350)	–	Het (NA)	LP (PP3, PM2, PM5, PM1, PP2, PP5, PS3)
CP_P42.1	*PIK3R2*	NM_005027.4: c.1117G > A (p. Gly373Arg)	–	Missense	Megalencephaly-polymicrogyria-polydactyly-hydrocephalus syndrome 1 (MIM #603387)	–	Het (NA)	LP (PM2, PS4, PM1, PP5)
CP_P51.1	*MFN2*	NM_014874.4: c.743 T > C (p. Leu248Pro)	–	Missense	Charcot–Marie–Tooth disease, axonal, type 2A2A (MIM #609260) Hereditary motor and sensory neuropathy VIA (MIM #601152)	–	Het (de novo)	LP (PM1, PP2, PM2, PM5, PP3, PM6)
CP_P53.1	*GRIN2B*	NM_000834.5:c.2021C > T (p. Pro674Leu)	–	Missense	Developmental and epileptic encephalopathy 27 (MIM #616139) Intellectual developmental disorder, autosomal dominant 6, with or without seizures (MIM #613970)	–	Het (de novo)	LP (PM1, PM2, PP2, PM6)
CP_P56.1	*SPAST*	NM_014946.4:c.1496G > T (p. Arg499Leu)	6.222e-7	Missense	Spastic paraplegia 4, autosomal dominant (MIM #182601)	–	Het (maternal)	LP (PM1, PM2, PM5, PP2, PP3, PP5)

Het, Heterozygous; Hem, hemizygous; Hom, Homozygous; P, Pathogenic; LP, Likely pathogenic; NA, Not applicable.

In an additional 17 patients (25.8%), variants of uncertain significance (VUS) were identified. Although these variants did not meet ACMG criteria for pathogenic or likely pathogenic classification, they were considered clinically relevant candidate variants due to strong genotype–phenotype concordance, segregation data when available, and consistency with previously reported disease mechanisms. These cases were therefore not regarded as definitive molecular diagnoses but were reported separately as putative genetic findings (Table 3).

**Table 3:** Variants of uncertain significance (VUS) identified in the cohort and clinical and genetic information of patients harboring these variants.

Patient ID	Gene	Variant	GnomAD v4 allele Freq.	Variant type	DISORDER (MIM)	CP risk factors	Zyg (segregation)	ACMG classification
CP_P15.1	*CLIC2*	NM_001289.6: c.103C > T (p. Arg35Cys)	0.00001	Missense	Intellectual developmental disorder, X-linked syndromic 32 (MIM #300886)		Hem (NA)	VUS (PM2)
CP_P58.1	*KCNT1*	NM_020822.3:c.3571G > A (p. Glu1191Lys)	0.000004	Missense	Developmental and epileptic encephalopathy 14 (MIM #614959)	–	Hom (maternal paternal)	VUS (PM2)
CP_P3.1	*GATM*	NM_001482.3: c.1081A > G (p. Asn361Asp)	–	Missense	Cerebral creatine deficiency syndrome 3 (MIM #612718)	–	Hom (NA)	VUS (PM2, PP3)
CP_P4.1	*GRM1*	NM_001278064.2: c.1124G > A (p. Arg375Gln)	6.196e-7	Missense	Spinocerebellar ataxia, autosomal recessive 13 (MIM #614831)	–	Hom (NA)	VUS (PM2, PP3)
CP_P28.1	*NDUFS3*	NM_004551.3:c.252A > T (p. Leu84Phe)	0.00002	Missense	Mitochondrial complex I deficiency, nuclear type 8 (MIM #618230)	–	Hom (NA)	VUS (PM2, PP3)
CP_P29.1	*CHKA*	NM_001277.3:c.630G > C (p. Pro210=)	6.196e-7	Splice site	Neurodevelopmental disorder with microcephaly, movement abnormalities, and seizures (MIM #620023)	–	Hom (maternal paternal)	VUS (PM2, PP3)
CP_P37.1	*ST3GAL5*	NM_003896.4: c.722G > C (p. Arg241Thr)	–	Missense	Salt and pepper developmental regression syndrome (MIM #609056)	–	Hom (maternal paternal)	VUS (PM2, PP3)
CP_P50.1	*KCNQ2*	NM_172107.4:c.1526-3C > A	6.209e-7	Splice site	Developmental and epileptic encephalopathy 7 (MIM #613720)	Neonatal seizures	Hom (maternal paternal)	VUS (PM2, PP3)
CP_P1.1	*SCN2A*	NM_001040142.2: c.5726C > T (p. Ala1909Val)	0.000001	Missense	Developmental and epileptic encephalopathy 11 (MIM #613721)	–	Het (NA)	VUS (PM2, PP3, PP2)
CP_P35.1	*CACNA1G*	NM_018896.5:c.4987G > C (p. Val1663Leu)	0.000002	Missense	Spinocerebellar ataxia 42 (MIM #616795) Spinocerebellar ataxia 42, early-onset, severe, with neurodevelopmental deficits (MIM #618087)	–	Het (NA)	VUS (PM2, PP3, PP2)
CP_P38.1	*CACNA1G*	NM_018896.5: c.1211G > A (p. Arg404Gln)	0.000001	Missense	Spinocerebellar ataxia 42 (MIM #616795) Spinocerebellar ataxia 42, early-onset, severe, with neurodevelopmental deficits (MIM #618087)	–	Het (NA)	VUS (PM2, PP3, PP2)
CP_P25.1	*KCNT1*	NM_020822.3:c.2756C > T (p. Thr919Met)	–	Missense	Developmental and epileptic encephalopathy 14 (MIM #614959)	–	Het (NA)	VUS (PM2, PP3, PM1)
CP_P6.1	*SCN8A*	NM_014191.4: c.3352G > C (p. Asp1118His)	–	Missense	Cognitive impairment with or without cerebellar ataxia (MIM #614306) Developmental and epileptic encephalopathy 13 (MIM #614558)	–	Het (NA)	VUS (PM2, PP2)
CP_P36.1	*PACS1*	NM_018026.4:c.1775G > A (p. Cys592Tyr)	–	Missense	Schuurs-Hoeijmakers syndrome (MIM #615009)	Neonatal seizures	Het (NA)	VUS (PM2, PP2)
CP_P23.1	*ATP1A2*	NM_000702.4:c.736A > G (p. Asn246Asp)	0.00001	Missense	Alternating hemiplegia of childhood 1 (MIM #104290) Developmental and epileptic encephalopathy 98 (MIM #619605)	–	Het (NA)	VUS (PM2, PP2, PP3)
CP_P18.1	*PNPT1*	NM_033109.5:c.2212C > T(p. Arg738Cys)	0.00001	Missense	Spinocerebellar ataxia 25 (MIM #608703)	Low birth weight	Het (NA)	VUS (PM2, PM5)
CP_P7.1	*ACY1*	NM_000666.3:c.1057C > T (p. Arg353Cys)	0.003	Missense	Aminoacylase 1 deficiency (MIM #609924)	–	Hom (NA)	VUS (BS1, PP5)

Het, Heterozygous; Hem, hemizygous; Hom, Homozygous; VUS, Variant of Uncertain Significance; NA, Not applicable.

**Figure 3:**
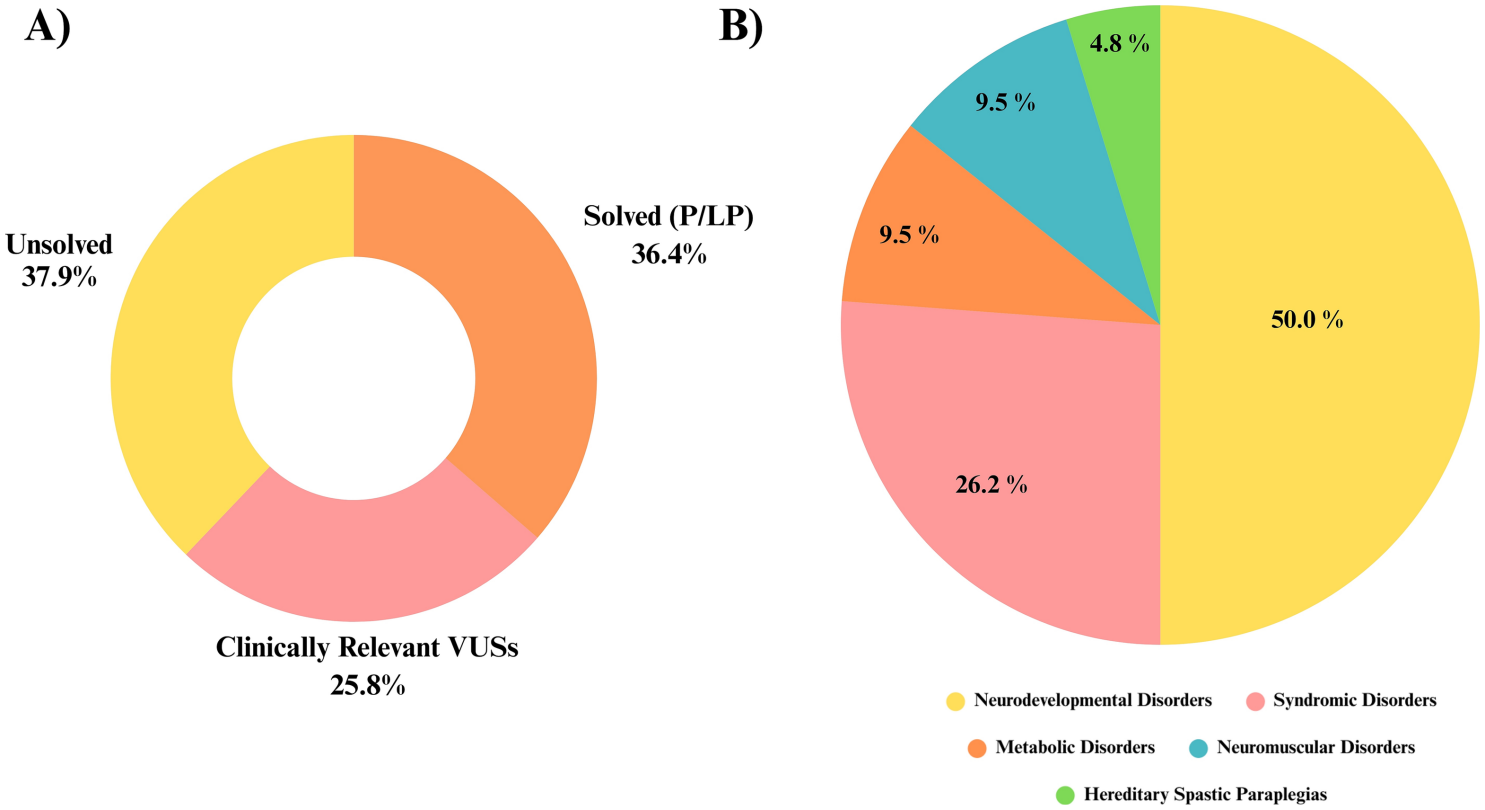
Genetic diagnosis status and distribution of disorder groups. **(A)** Genetic diagnostic results in the CP patient group. “Solved (P/LP)” indicates cases with pathogenic or likely pathogenic variants (36.4%), while “Clinically relevant VUSs” includes cases with variants of uncertain significance (25.8%). The remaining 37.9% of cases remain genetically unresolved. **(B)** Distribution of disease categories among genetically diagnosed cases. The largest group is Neurodevelopmental Disorders (50.0%), followed by Syndromic Disorders (26.2%), Metabolic Disorders (9.5%), Neuromuscular Disorders (9.5%), and Hereditary Spastic Paraplegias (4.8%).

When considered together, pathogenic/likely pathogenic variants and clinically relevant VUS accounted for molecular findings in 41 of 66 patients (62.1%), reflecting the overall genetic landscape of the cohort. However, only cases harboring P/LP variants were classified as having a definitive genetic diagnosis, while cases with VUS remain under ongoing evaluation (Figure 3A).

In total, 42 variants were identified across these 41 individuals. Missense variants constituted the majority (66.6%, 28/42), followed by truncating variants including frameshift and stop-gain variants (26.2%, 11/42), and canonical splice-site variants (7.1%, 3/42). No pathogenic mitochondrial DNA variants or clinically relevant copy number variants were detected in this subset.

Among the 42 genetic diagnoses in 41 solved cases, autosomal dominant inheritance was most common (59.5%), followed by autosomal recessive (35.7%, all homozygous) and X-linked (4.76%). One patient had dual diagnoses involving distinct phenotypes.

Patients were classified into five phenotype-based groups based on their underlying molecular etiologies. Neurodevelopmental disorders were the most frequent (50.0%), followed by syndromic disorders (26.2%), neuromuscular disorders (9.5%), metabolic disorders (9.5%), and hereditary spastic paraplegias (4.8%) (Figure 3B).

The genes identified as harboring potential disease susceptibility variants in the Turkish patient cohort were analyzed for network and pathway enrichment using the STRING web tool (v12.0). The STRING network analysis of these genes yielded a PPI enrichment *p*-value of 9.08 × 10^−13^, indicating that at least a part of the proteins is functionally related and likely to participate in shared biological pathways or processes (Supplementary Figure 2).

Gene Ontology enrichment revealed strong over-representation of neurophysiological processes, including *membrane depolarization during action potential* (FDR = 1.34 × 10^−13^) and *glutamatergic synaptic transmission* (FDR = 2.46 × 10^−12^) (Supplementary Figure 3; Supplementary Table 3). Molecular function analysis highlighted *voltage-gated ion channel activity* (FDR = 1.85 × 10^−13^) and *metal ion transmembrane transporter activity* (FDR = 5.47 × 10^−12^) (Supplementary Figure 4; Supplementary Table 4).

Cellular component terms were dominated by neuronal and membrane-associated structures, such as *neuron projection* (FDR = 2.93 × 10^−14^), *synapse* (FDR = 1.07 × 10^−13^), and *voltage-gated sodium channel complex* (FDR = 2.85 × 10^−12^) (Supplementary Figure 5; Supplementary Table 5). KEGG pathway analysis identified enrichment in *cAMP signaling* (FDR = 3.21 × 10^−5^), *Ras signaling* (FDR = 7.62 × 10^−5^), and *thyroid hormone signaling* (FDR = 1.34 × 10^−4^) (Supplementary Table 6; Supplementary Figure 6). Disease enrichment analysis linked the prioritized genes most strongly to *epilepsy* (FDR = 5.21 × 10^−16^), *autism spectrum disorder* (FDR = 8.14 × 10^−15^), and *intellectual disability* (FDR = 3.66 × 10^−14^) (Supplementary Table 7; Supplementary Figure 7).

**Figure 4:**
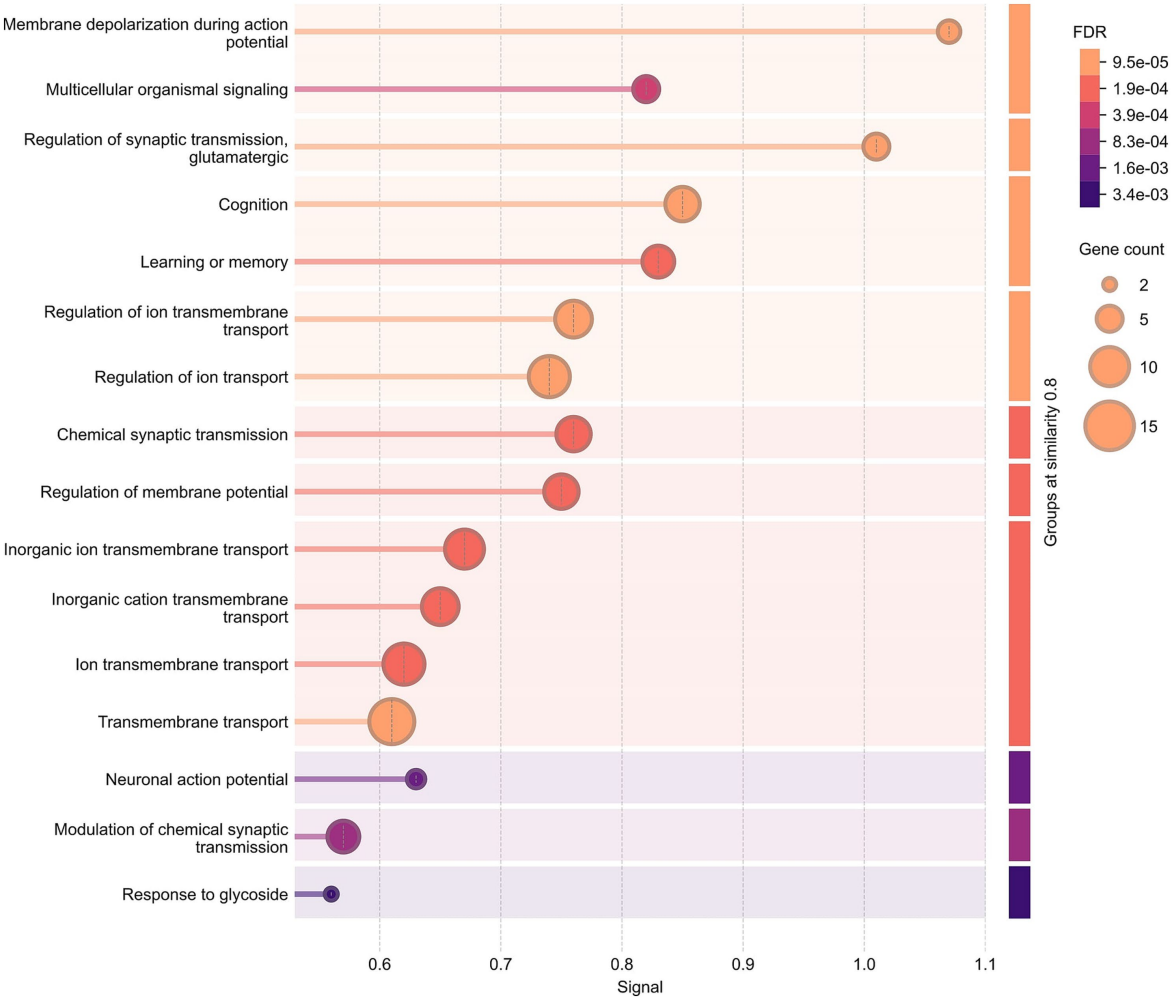
Functional enrichment analysis of prioritized genes in the cohort. Biological process (gene ontology) enriched in analysis conducted in STRING web tool (v12.0). The plot displays the top 16 enriched GO terms ranked by the highest signal scores and lowest FDR values, grouped by semantic similarity (threshold ≥0.8). Signal is a composite measure prioritizing enriched terms based on statistical significance (adjusted *p*-value) and informativeness. While the strength score (Supplementary material) represents the effect size, calculated as the log₁₀ ratio of observed to expected gene counts. Each bar corresponds to the signal magnitude for an individual GO term, and the bubbles at the end of each bar denote the number of genes in the query set that overlap with genes annotated with that GO term. Statistical significance of each overlap is assessed using a hypergeometric test, and *p*-values are corrected for multiple testing using the Benjamini–Hochberg method, reported as FDR (false discovery rate). A GO term with a lower signal may still appear higher in the plot if it is semantically grouped with a higher-ranking term. Background shading reflects the FDR of each group.

Taken together, these findings support a mechanistic link between the selected variants and central nervous system dysfunction, particularly processes affecting ion homeostasis, neuronal excitability, and neurodevelopmental pathologies (Figure 4 and all raw data are provided in Supplementary material).

## Discussion

Genomic testing is transforming the diagnostic landscape of CP, revealing that many cases previously considered idiopathic have an underlying genetic basis. CP includes a wide range of motor and neurodevelopmental impairments, yet identifying a clear genetic cause remains difficult in many instances. In our study, we highlight the transformative role of genomic analyses in uncovering underlying molecular mechanisms, which pave the way for personalized treatment strategies, informed genetic counseling, and tailored long-term follow-up.

In this study, the use of advanced bioinformatics pipelines combined with deep phenotyping and diverse genomic sequencing approaches yielded a diagnostic rate of approximately 38%, enabling the identification of genetic etiologies in previously undiagnosed CP patients. Reported diagnostic yields in prior genomic studies range from 24 to 58%, influenced by factors such as the inclusion of idiopathic cases, the presence of neurodevelopmental comorbidities, and the use of trio-based sequencing strategies (10, 15, 16, 36).

Our high diagnostic yield is likely due to several factors: careful patient selection based on strict inclusion and exclusion criteria, the use of comprehensive deep phenotyping, and the deliberate exclusion of individuals with CP clearly linked to non-genetic causes (e.g., perinatal asphyxia, extreme prematurity). Additionally, integrating advanced bioinformatics pipelines with thorough segregation analyses enhanced our ability to identify genetic causes that had previously gone undetected in this patient group. CP shares significant phenotypic overlap with other neurodevelopmental disorders, and many genetic neurological conditions can resemble CP because of similar motor and developmental features (37). In our cohort, the variety of underlying genetic causes underscored the diverse molecular mechanisms involved in CP and CP-like presentations. Reverse phenotyping helped refine diagnoses into five main groups (Tables 2, 3), with neurodevelopmental disorders, particularly epileptic encephalopathies [e.g., *WWOX* (MIM: 605131), *ATP1A3* (MIM: 619606)] and spinocerebellar ataxia [e.g., *PNPT1* (MIM: 610316)], forming the largest subgroup. Variants in genes such as *L1CAM* (MIM: 304840), *CTNNB1* (MIM: 116806), and *SYNGAP1* (MIM: 603384), recently suggested as candidates in CP pathophysiology (36), are involved in neuronal migration, synaptogenesis, and axonal guidance (1, 38). These findings support the idea that CP and related neurodevelopmental disorders may exist on a shared clinical and molecular spectrum, highlighting the importance of genomic testing in clarifying diagnostic boundaries where phenotypic overlap complicates classification.

Although all patients presented with at least one reported comorbidity, these features should not necessarily be interpreted as independent conditions co-occurring with Cerebral Palsy. In many cases, they represent core manifestations of the underlying genetic disorder. For example, epilepsy in patients with developmental and epileptic encephalopathies constitutes a primary disease feature rather than a secondary comorbidity. Similarly, visual impairment in *CTNNB1*-related (MIM: 116806) disorders and ataxia in *CACNA1G*-associated (MIM: 604065) conditions are well-established components of their respective phenotypic spectrum.

The second largest group comprised syndromic cases, where dysmorphic features especially in patients with severe neurodevelopmental impairments were often overlooked under an initial CP diagnosis (39). Such oversight can lead to misclassification and missed phenotypic clues essential for accurate genetic evaluation. In our study, incomplete phenotyping and underreporting of HPO terms contributed to missed variants in syndromic genes during initial panel-based analyses (40). Reverse phenotyping later revealed unrecognized dysmorphic features, such as those associated with *ARID1B* (Coffin–Siris syndrome, MIM: 614556) and *PPM1D* (Jansen–de Vries syndrome, MIM: 617450) (16). We recommend thorough dysmorphic assessments with comprehensive HPO annotation, ideally performed by clinical geneticists before testing, to capture subtle yet diagnostically relevant traits and improve yield in CP and CP-like presentations. Beyond increasing diagnostic yield, reverse phenotyping also facilitated genotype–phenotype expansion, allowing the identification of novel or atypical clinical manifestations linked to known disease genes, thereby refining their phenotypic spectra.

. The third group comprised metabolic disorders, identified in four patients with variants in *GATM* (MIM: 612718), *ACY1* (MIM: 609366), *NDUFS3* (MIM: 606934), and *NGLY1* (MIM: 615273). These genes are associated with treatable or manageable conditions that often present with CP-like features, including developmental delay, hypotonia, seizures, and progressive motor impairment. As highlighted in a systematic review by Leach et al., more than 50 inborn errors of metabolism can mimic CP, underscoring the value of early metabolic and genetic assessment to prevent irreversible neurological damage (41). Our findings support this, showing that integrating biochemical screening with genomic testing facilitates timely diagnosis, enables targeted interventions, and can significantly improve clinical outcomes in CP-like presentations.

The fourth group comprised patients with neuromuscular phenotypes, including congenital myopathies and hereditary motor neuropathies, with pathogenic variants in *MFN2* (MIM: 608507), *PLA2G6* (MIM: 603604), and *COL6A1* (MIM: 120220) identified in four individuals. The patient (CP_P51.1) with an *MFN2* variant presented with spastic paraparesis and brisk deep tendon reflexes, yet the overall presentation was more consistent with *MFN2*-related axonal neuropathy (MIM: 609260, 601,152). The *PLA2G6* variant was detected in an individual (CP_P2.1) with motor delay, seizures, and structural brain anomalies, including corpus callosum hypoplasia and ventriculomegaly, features that overlap with, but are not typical of, CP. Other patients (CP_P8.1 and CP_P30.1) initially diagnosed with CP who exhibited neonatal hypotonia, delayed ambulation, and joint contractures were later found to harbor a *COL6A1* variant, consistent with collagen VI-related muscular dystrophy (MIM: 158810, 254,090). These cases illustrate the phenotypic convergence between certain neuromuscular disorders and CP, particularly in the absence of perinatal risk factors or with atypical disease progression, and underscore the importance of genomic investigation for accurate diagnostic classification (42).

The fifth subgroup in our cohort encompassed hereditary spastic paraplegias (HSPs), a clinically and genetically heterogeneous group of neurodegenerative disorders defined by progressive lower-limb spasticity and gait impairment. Early-onset forms pose a particular diagnostic challenge, as hallmark motor features such as spasticity and delayed motor milestones substantially overlap with those of spastic Cerebral Palsy (CP), frequently resulting in initial misclassification (43). Among the HSP-associated genes, *SPAST* (MIM: 604277) and *KIF1A* (MIM: 601255) collectively account for over 40% of autosomal dominant HSP cases (44). *SPAST* encodes spastin, a microtubule-severing enzyme crucial for axonal growth, maintenance, and neuronal connectivity (45). Pathogenic *SPAST* variants demonstrate pronounced intrafamilial and interfamilial variability in age of onset, clinical severity, and disease trajectory, thereby complicating recognition (46). In our series, CP_P56.1 harbored pathogenic *SPAST* variants, each with a distinct phenotypic profile, despite sharing the same molecular diagnosis as the mother. *KIF1A* encodes an anterograde motor protein essential for the axonal transport of membranous organelles (47). One patient in our cohort, initially diagnosed with CP based solely on clinical presentation, was ultimately found to carry a pathogenic *KIF1A* variant. Notably, *KIF1A*-related disorders span a spectrum from HSP to complex neurodevelopmental phenotypes, including CP-like presentations with cognitive impairment and seizures (40). A defining distinction between HSP and CP lies in the progressive nature of spasticity in HSP, underscoring the necessity of genomic testing to accurately delineate these entities and guide prognosis, surveillance, and therapeutic strategies (48).

STRING network analysis showed a significant enrichment, indicating that the identified genes are not randomly distributed but functionally connected within shared biological modules. GO enrichment highlighted biological processes such as ion transport, membrane depolarization, and synaptic signaling, together with molecular functions related to voltage-gated ion channel activity and metal ion transport (49) (Supplementary Figure 2). Cellular component terms emphasized neuron projections, synapses, and ion channel complexes, supporting their localization to neuronal structures (Supplementary Figure 4). KEGG pathway analysis further revealed enrichment in cAMP, Ras, and thyroid hormone signaling, linking the candidate genes to key regulatory cascades (Supplementary Figure 5). Collectively, these convergent results suggest that disruption of ion homeostasis and neuronal excitability represents a central mechanism, consistent with disease enrichment findings that strongly associate these genes with epilepsy, autism spectrum disorder, and intellectual disability.

When interpreting our results, several limitations should be considered. The use of heterogeneous sequencing platforms (CES, WES, and WGS) affected the resolution for detecting certain variant types, particularly copy number variants and mitochondrial DNA variants. The relatively small WGS subset further limited genome-wide variant detection, while CNV analysis was constrained by the resolution of WES data. In addition, clinical and phenotypic data were collected retrospectively, and Human Phenotype Ontology (HPO) terms were extracted from hospital-based records, which may have resulted in incomplete phenotypic annotation and limited genotype–phenotype correlations. The enrichment of genetically suspected cases may also have introduced selection bias. Finally, the modest cohort size limits the generalizability of our findings, underscoring the need for larger, multicenter studies integrating trio-based WES/WGS, high-resolution CNV detection, deep phenotyping, and functional validation of clinically relevant variants to improve diagnostic yield and precision.

In conclusion, our findings underscore the pivotal role of genomic testing in refining the diagnosis of CP and CP-like presentations, revealing a broad spectrum of underlying genetic etiologies that often extend beyond traditional clinical boundaries. In line with the International Cerebral Palsy Genomics Consortium (ICPGC) consensus, when a pathogenic or likely pathogenic variant is identified in a patient whose phenotype fulfills the consensus definition of CP, the diagnosis should be retained rather than reclassified as a mimic, highlighting the need to integrate genetic findings into, rather than replace, clinical classification. By integrating advanced sequencing technologies, reverse phenotyping, and deep clinical characterization, we not only increased diagnostic yield but also expanded the known genotype–phenotype spectrum of several disease genes, including many not previously associated with CP. These results highlight the importance of considering genomic evaluation early in the diagnostic pathway, particularly for patients with atypical features or without clear perinatal risk factors, to enable timely and targeted interventions. Moving forward, larger multicenter studies employing standardized trio-based WES/WGS, high-resolution CNV analysis, and systematic phenotyping will be essential for fully delineating the genetic architecture of CP, improving diagnostic precision, and ultimately guiding personalized management strategies for affected individuals.

## Data Availability

The original contributions presented in the study are included in the article/Supplementary material, further inquiries can be directed to the corresponding authors.

## Footnotes

1
https://github.com/GenivaInformatics/gennext-workflows

2
https://panelapp-aus.org/panels/73/

3
https://string-db.org/

4
http://www.genome.jp/kegg
