# Supplementary material for: Unmasking genetic etiologies in neurodevelopmental disorders characterized by Cerebral Palsy: insights from integrative genomic approaches
Source: Front Neurol. 2026 Feb 23;17:1742186. doi: 10.3389/fneur.2026.1742186 (PMC12979860; doi:10.3389/fneur.2026.1742186)
Supplement: Supplementary file 1 [file Supplementary_file_1.docx]

**SUPPLEMENTARY INFO**

***Supplementary Table 1. Clinical Characteristics of CP cohort***

| **Number of individuals** | 66 |
| --- | --- |
| **Age** |  |
| Range | 3 m - 18y |
| Mean age | 6 y |
| 0-2 (≤2) | 24.24% (16/66) |
| 2-5 (>2-≤5) | 19.69% (13/66) |
| 5-12 (>5-≤12) | 0.48% (32/66) |
| 12-18 (>12-≤18) | 6.06% (4/66) |
| **Sex (%)** |  |
| Male | 51.51% (34/66) |
| Female | 48.48% (32/66) |
| **Consanguinity** |  |
| Consanguineous parents | 43.9% (29/66) |
| Non-consanguineous parents | 56.1% (37/66) |

***Supplementary Table 2. Most Frequent Found Disorders & Mutated Genes***

| ***Gene*** | **# of Cases** | **Inheritance** | **OMIM ID** | **DISORDER** |
| --- | --- | --- | --- | --- |
| *SCN2A* | 1 | AD | 182390 | Developmental and epileptic encephalopathy 11 Episodic ataxia, type 9 Seizures, benign familial infantile 3 |
| *PLA2G6* | 1 | AR | 603604 | Infantile neuroaxonal dystrophy Neurodegeneration with brain iron accumulation 2B Parkinson disease 14, autosomal recessive |
| *GATM* | 1 | AR | 602360 | Cerebral creatine deficiency syndrome 3 |
| *GRM1* | 1 | AR | 604473 | Spinocerebellar ataxia, autosomal recessive 13 |
| *SCN8A* | 1 | AD | 600702 | Myoclonus, familial, 2 Cognitive impairment with or without cerebellar ataxia Developmental and epileptic encephalopathy 13 Seizures, benign familial infantile,5 |
| *ACY1* | 1 | AR | 104620 | Aminoacylase 1 deficiency, AR |
| *COL6A1* | 2 | AR | 120220 | Ulrich congenital muscular dystrophy 1A |
| *ARID1B* | 1 | AD | 614556 | Coffin-Siris syndrome 1, AD |
| *WWOX* | 1 | AR | 605131 | Developmental and epileptic encephalopathy 28 Spinocerebellar ataxia, autosomal recessive 12 |
| *CLIC2* | 1 | XLR | 300138 | Intellectual developmental disorder, X-linked syndromic 32 |
| *ASXL1* | 1 | AD | 612990 | Bohring-Opitz syndrome |
| *SETBP1* | 1 | AD | 611060 | Intellectual developmental disorder, autosomal dominant 29 Schinzel-Giedion midface retraction syndrome |
| *SYNGAP1* | 1 | AD | 612621 | Intellectual developmental disorder, autosomal dominant 5 |
| *PNPT1* | 1 | AD | 610316 | Spinocerebellar ataxia 25 |
| *CTNNB1* | 1 | AD | 116806 | Exudative vitreoretinopathy 7 Neurodevelopmental disorder with spastic diplegia and visual defects |
| *ATP1A2* | 1 | AD | 182340 | Alternating hemiplegia of childhood 1 Developmental and epileptic encephalopathy 98 |
| *CHKA* | 1 | AR | 118491 | Neurodevelopmental disorder with microcephaly, movement abnormalities, and seizures |
| *KCNT1* | 2 | AD, AR? | 608167 | Developmental and epileptic encephalopathy 14 |
| *NSUFS3* | 1 | AR | 603846 | Mitochondrial complex I deficiency, nuclear type 8 |
| *SAMD9* | 1 | AD | 610456 | MIRAGE syndrome |
| *COL1A2* | 1 | AD | 120160 | Combined osteogenesis imperfecta and Ehlers-Danlos syndrome 2 Ehlers-Danlos syndrome, arthrochalasia type,2  Osteogenesis imperfecta, type II,III,IV |
| *CACNA1G* | 2 | AD | 604065 | Spinocerebellar ataxia 42 Spinocerebellar ataxia 42, early-onset, severe, with neurodevelopmental deficits |
| *PACS1* | 1 | AD | 607492 | Schuurs-Hoeijmakers syndrome |
| *ST3GAL5* | 2 | AR | 604402 | Salt and pepper developmental regression syndrome |
| *PPM1D* | 1 | AD | 605100 | jansen-de Vries syndrome |
| *LINS1* | 1 | AR | 610350 | Intellectual developmental disorder, autosomal recessive 27 |
| *PIK3R2* | 1 | AD | 603157 | Megalencephaly-polymicrogyria-polydactyly-hydrocephalus syndrome 1 |
| *CACNA1E* | 1 | AD | 618285 | Developmental and epileptic encephalopathy 69, AD |
| *PTEN* | 1 | AD | 601728 | Macrocephaly / autism syndrome |
| *L1CAM* | 1 | XLR | 308840 | Corpus callosum, partial agenesis of Hydrocephalus, congenital, X linked MASA syndrome |
| *SPAST* | 1 | AD | 604277 | Spastic paraplegia 4, autosomal dominant |
| *MFN2* | 1 | AD | 608507 | Charcot-Marie-Tooth disease, axonal, type 2A2A Hereditary motor and sensory neuropathy VIA |
| *KCNQ2* | 1 | AR? | 602235 | Developmental and epileptic encephalopathy 7, AD |
| *GRIN2B* | 1 | AD | 138252 | Developmental and epileptic encephalopathy 27, AD |
| *KIF1A* | 1 | AD | 601255 | NESCAV syndrome, AD Spastic paraplegia 30, autosomal dominant |
| *NGLY1* | 1 | AR | 610661 | *Congenital disorder of deglycosylation 1, AR* |
| *ATP1A3* | 1 | AD | 182350 | *Developmental and epileptic encephalopathy 99, AD* |
| *STXBP1* | 1 | AD | 602926 | *Developmental and epileptic encephalopathy 4, AD, AR* |

**Medical History of Patients**

**CP_P1.1**: A 30-month-old female presented with global developmental delay, hand tremor, and gait imbalance. She was born with aortic valve stenosis. Brain MRI showed cerebral atrophy, cerebellar vermis hypoplasia, and periventricular white matter hyperintensities. Dysmorphic features included a broad forehead, anteverted nares, and a short nasal bridge. There is no other affected family member; the parents were first cousins.

**CP_P2.1:** A male patient was diagnosed with cerebral palsy and global developmental delay in the neonatal period. Brain MRI revealed corpus callosum hypoplasia, frontotemporal cerebral atrophy, and globus pallidus calcification. He also presented with gastroesophageal reflux and feeding difficulties. Parental consanguinity was present, as the parents are first cousins, and there were no other affected family members.

**CP_P3.1:** A 11-year-old female patient with moderate global developmental delay. No further clinical information is available.

**CP_P4.1**: A 11-year-old male patient who has been followed up with mental motor retardation since birth. Parents were first-degree cousins. Karyotype analysis was normal.

**CP_P5.1:** A female patient was born as the first child (G1P1) at 36 gestational weeks, weighing 2330 grams and measuring 42 cm, to a 26-year-old mother. The neonatal period was uneventful until 34 weeks; however, after 39 weeks, feeding difficulties were noted. She was observed to be small for gestational age. Head control was achieved on time, but at 6 months, a delay in sitting was recognized, along with delayed attainment of other developmental milestones. Her sleep pattern is irregular, with frequent awakenings. She has features of ADHD and autistic behavior. Cranial MRI was normal in 2015, but cerebral atrophy was detected in 2019. On physical examination, she presented with relative macrocephaly, tremors, and an ataxic gait. No causal variant was detected in CES reanalysis.

**CP_P6.1:** A female patient was born at term (G1P1) to a 25-year-old mother, weighing 4135 grams. Prenatal follow-up was unremarkable, including non-invasive screening and double test performed due to increased nuchal thickness. The perinatal period was uneventful, and no intensive care follow-up was required. Hypotonia was noted at the second postnatal week, leading to ongoing neurology follow-up. There is no history of seizures, but she has global developmental delay and continues to receive physical therapy and special education. There is no known parental consanguinity.

**CP_P7.1:** A female patient presented with hypotonia noted at 2.5 months of age and has been followed since then. Seizures began at 5 months old and were subsequently controlled with anti-seizure medication. Upon examination, the lower extremities exhibited a more pronounced increase in tone, and serum creatinine levels were frequently elevated. Distal tapering was observed in the hands, and she displayed dysmorphic facial features. Speech and language development were delayed, and she remains non-ambulatory. There is second-degree parental consanguinity.

**CP_P8.1:** The patient is being followed for distal arthrogryposis. No further clinical information is available.

**CP_P9.1:** A male patient was born to a 32-year-old mother at 39 gestational weeks (G2P2), with a birth weight of 3550 grams. The pregnancy was regularly followed and no complications were noted during the prenatal period. The patient experienced perinatal asphyxia during delivery and required intensive care for three months. He achieved speech and walking milestones at age 3. He attends special education and, as an inclusive student, has learned to read and write. The patient has been hospitalized frequently due to infections and reflux, and underwent surgery for cryptorchidism. There is no known parental consanguinity, but there are similar cases of mental and physical disabilities in the extended family. No causal variant was detected in CES reanalysis.

**CP_P10.1:** The patient was followed for polyneuropathy and hypotonia. During the perinatal period, she required intensive care for two months due to respiratory distress. She exhibited a weak sucking reflex and difficulty swallowing. The patient passed away at 4 months of age. There is first-degree consanguinity between the parents. Previous microarray analysis and CES reanalysis did not identify any causal genetic alterations.

**CP_P11.1:** Male patient with microcephaly and global developmental delay. No causal genetic alterations were detected previous microarray analysis and CES reanalysis.

**CP_P12.1:** A male patient was born at term (G2P2) to a 28-year-old mother, with a birth weight of 2800 grams. He achieved head control at 4–5 months and was able to sit without support after one year of age. His speech is limited to single words. Seizures began on the first postnatal day but were controlled with levetiracetam, and he has been seizure-free for the past year. There are no other known health problems. There is no known parental consanguinity.

**CP_P13.1:** A female patient was born as the fifth child (G5P5) via cesarean section to a 28-year-old mother, with a birth weight of 3500 grams. She did not require neonatal intensive care. Hypotonia was present but mild until 6 months of age, after which motor delay became more apparent. There were no feeding problems during the first six months; however, the mother reported that the patient was unusually quiet, never cried, and was always calm. There was no history of social smiling or recognizing the mother.

Bruxism and hyperacusis were noted. The first seizure occurred at 8 years old and is currently controlled with medication. Cranial MRI showed increased T2 signal in the periventricular area. Electroencephalography (EEG) revealed a local epileptiform anomaly in the frontocentral region. On physical examination, spasticity was observed in all extremities, particularly in the lower limbs, with limited extension at the knees and elbows. No causal variant was detected in CES or microarray analysis.

**CP_P14.1:** A female patient was born as the third child (G3P3) at term via vaginal delivery, weighing 3620 grams. The pregnancy was regularly followed and complicated by intrauterine growth retardation. There was no need for postnatal intensive care. Tonic-clonic seizures began at two weeks of age, and anti-seizure medication was initiated. She is unable to sit independently and presents with spasticity and significant kyphoscoliosis. No causal variant was detected in initial CES or microarray analysis. The patient’s brother died with a diagnosis of Coffin-Siris syndrome. There is first-degree consanguinity between the parents.

**CP_P15.1:** A male patient was born at 38 weeks of gestation via vaginal delivery to a 40-year-old mother (G4P4), with a birth weight of 4000 grams and length of 52 cm. Following birth, he required intensive care due to neonatal contractions. He has no history of seizures. He can walk with assistance, but head control was not achieved until after one year, and he has not yet developed speech. Cranial MRI revealed hydrocephalus, cerebral atrophy, and corpus callosum hypoplasia. On physical examination, spasticity was prominent in all extremities. No causal variant was detected in the initial CES or microarray analyses. There is no parental consanguinity.

**CP_P16.1:** A male patient was born by cesarean section at 38 weeks of gestation to a 23-year-old mother (G3P1). Prenatal follow-up revealed corpus callosum agenesis, and pregnancy termination was recommended. In the postnatal period, the patient required intensive care monitoring. He presented with general growth retardation, delayed speech, and confirmed corpus callosum agenesis. There is no known parental consanguinity. Previous microarray analysis and CES reanalysis did not detect any causal genetic alterations.

**CP_P17.1:** A male patient (G2P2) was born at 35+2 weeks of gestation with a birth weight of 1400 grams. He demonstrated delayed developmental milestones, signs of sensorineural hearing loss, and mild intellectual disability. Pediatric nephrology evaluation revealed proteinuria, thrombocytopenia, and hematuria. There is no parental consanguinity. Initial CES and microarray analyses did not identify any causal variant.

**CP_P18.1:** A male patient was born to a 20-year-old mother (G2P2) as the first living child via cesarean section at 38 weeks of gestation, weighing 2470 grams. He experienced frequent infections and recurrent fevers. There was a delay in sitting, walking, and speaking milestones. The patient was followed for neuromotor developmental delay and epilepsy, and there were no similar cases in the family.

**CP_P19.1:** A female patient was born at term (G4P4) to a 30-year-old mother. She began experiencing seizures at five months of age and required anti-seizure medication for three years. She is currently seizure-free and is receiving special education. Her siblings are healthy, and there is no parental consanguinity. Previous microarray analysis and CES reanalysis did not identify any causal genetic alterations.

**CP_P20.1:** The patient was referred with a diagnosis of cerebral palsy and neuromotor developmental delay. There were no dysmorphic features observed. Previous microarray analysis and CES reanalysis did not detect any causal genetic alterations.

**CP_P21.1:** A male patient has been followed for mental-motor retardation, retinal detachment, and central hypotonia; however, detailed clinical data are not available.

**CP_P22.1:** A 7-year-old female patient with clinically diagnosed cerebral palsy and nephrotic syndrome.

**CP_P23.1:** A female patient presented with severe hypotonia at 4 months of age and has been monitored since then, initially with suspicion of a metabolic disorder. Convulsions began at 7 months of age. She displays atypical facial features and has not yet achieved head control or independent support. There was no parental consanguinity, and her siblings were healthy.

**CP_P24.1**: A male patient followed up with a diagnosis of neonatal hypotonia, microcephaly and global developmental delay. He also had a medical history of seizures.

**CP_P25.1:** The girl, who was followed up with the diagnosis of cerebral palsy, has also microcephaly, seizures, and motor retardation. There was a first-degree consanguinity between the parents.

**CP_P26.1:** The female patient was born as G1P1 from a 26-year-old mother at 29 weeks of age by cesarean section of 1613 gr. She was followed in the neonatal intensive care unit due to respiratory distress, feeding difficulties, joint contracture and hip dislocation.

**CP_P27.1:** A male patient was born from a 28-year-old mother at 36 weeks. Due to respiratory distress in the perinatal period, he needed follow-up in the neonatal intensive care unit.He had global developmental delay. Hearing impairment was detected in the neonatal period. He has had gait inbalance. Eye examination revealed retinitis pigmentosa. There was no obvious dysmorphic findings. There was no known parental consanguinity. No causal genetic alterations were detected in CES reanalysis.

**CP_P28.1**: A male patient was born 3300 gr at term with a vaginal delivery as G3P2. He had global developmental delay, epilepsy, autism and absent speech. Parents were first-degree cousins.

**CP_P29.1:** The male patient was born from a 25y mother as G5P2, 2500 gr at term. He had neonatal hypotonia from birth. He had severe global developmental delay. He had his first seizure when he was 4 months old. The seizures continued in tonic-clonic type, 5-6 times a day, and the seizures continued despite multiple anti-seizure drugs. Cranial MRI revealed diffuse cerebral atrophy and striatal T2 hyperintensity. On physical examination, axial hypotonicity was prominent. Dysmorphic findings were as followed: Microcephaly, narrow forehead, anteverted ears, long eyelashes, depressed nasal bridge, and high-arched eyebrows. No causal variant was detected in the microarray and CES reanalysis.

**CP_P30.1:** A 5-year-old female patient was born at term via cesarean section due to breech presentation, with a birth weight of 2500 grams. She required a 12-day stay in the neonatal intensive care unit due to weak sucking, although no jaundice was observed. Hypotonia was noted at birth, and breastfeeding was initiated.

She presented with congenital myopathy, scoliosis, kyphosis, torticollis, developmental hip dysplasia, and distal joint hyperlaxity. Muscle biopsy revealed dystrophic changes. Denver developmental screening test showed delayed gross motor development. Hip ultrasonography demonstrated incomplete coverage of the femoral head by the acetabular labrum.

**CP_P31.1:** A female patient was born via vaginal delivery (G2P2), with a birth weight of 1400 grams and a length of 48 cm. There was no prenatal follow-up. She required neonatal intensive care monitoring in the postnatal period.

She was evaluated due to developmental delay, absence of speech, and delayed ambulation. On physical examination, strabismus, mild hypotelorism, high-arched narrow palate, and bilateral fifth finger clinodactyly were noted. Previous clinical exome sequencing and microarray analysis did not detect any causal genetic alterations. There is first-degree parental consanguinity.

**CP_P32.1 & CP_P32.2:** Two siblings (one female, one male) presented with neuromotor developmental delay and microcephaly. There is first-degree parental consanguinity. No causal variant was detected in the clinical exome sequencing or microarray analysis.

**CP_P33.1:** A male patient was born by cesarean section at 37+2 weeks of gestation. He required follow-up in the neonatal intensive care unit due to hypotonia and respiratory distress in the postnatal period. He has a healthy sibling. There is first-degree parental consanguinity. No causal variant was identified in previous genetic analyses.

**CP_P34.1:** A male patient has been followed for mental retardation and epilepsy. He was also being monitored by pediatric metabolism due to abnormalities in glutamine and homocysteine metabolism. No causal variant was detected in CES reanalysis.

**CP_P35.1:** A male patient was born via vaginal delivery at 43 weeks of gestation. There was no reported prenatal follow-up. In the neonatal period, he lacked a sucking reflex and did not cry. He was admitted to the neonatal intensive care unit due to feeding difficulties.

He underwent surgery for cryptorchidism. He is currently followed for spastic tetraparesis, epilepsy, microcephaly, cerebellar atrophy, and ongoing respiratory problems.

**CP_P36.1:** A female patient was born at 36 weeks of gestation. There was no history of perinatal asphyxia. She was followed in the neonatal intensive care unit due to seizures in the postnatal period, which have continued as drug-resistant.

She achieved unsupported sitting between 1.5–2 years of age. She is not yet able to walk and speaks only 2–3 syllable words. She also has visual problems, including strabismus and nystagmus. There is no known parental consanguinity.

**CP_P37.1:** A female patient was born to a 30-year-old mother (G3P3) with a birth weight of 3050 grams. She did not cry at birth and was admitted to the neonatal intensive care unit due to feeding difficulties.

The first seizure occurred at 6 months of age. She is unable to hold her head or sit, even with support. Generalized hypotonia has been present since birth. She has a documented cow’s milk allergy and does not make visual contact. Ophthalmologic evaluation revealed abnormal visual evoked potentials and electroretinography. No antiepileptic treatment has been initiated.

Prenatal follow-up was reported as uneventful. She has two healthy brothers and no family history of similar disease. On physical examination, a depressed nasal bridge and mild retrognathia were noted. Cranial MRI showed cerebral atrophy and marked neuromotor delay. There is no parental consanguinity.

**CP_P38.1:** A 7-year-old female patient has been followed with a pre-diagnosis of cerebral palsy, along with co-morbid autism and epilepsy.

**CP_P39.1:** A 7-year-old male patient was born at 34 weeks of gestation via vaginal delivery to a G2P2 mother, with a birth weight of 2160 grams. Prenatal follow-up was regular, but complicated by polyhydramnios and maternal bleeding. He was admitted to the neonatal intensive care unit due to postnatal complications and was diagnosed with neonatal sepsis.

Head control developed on time, but walking and speech milestones were delayed. There is no similar case in the family, and no known parental consanguinity.

**CP_P40.1:** A male patient was born as G1P1. He underwent surgery for atrial and ventricular septal defects. He is unable to walk or talk and is currently receiving special education. There is first-degree consanguinity between the parents.

**CP_P41.1:** A female patient has been followed for growth retardation and hypotonia. Despite a normal EEG, she experiences epileptic seizures. Cranial MRI revealed cerebral atrophy, inferior venous hypoplasia, dilation of the lateral ventricles, and widened cortical fissures. No causal variant was detected in CES reanalysis. There is parental consanguinity.

**CP_P42.1:** A female patient was born at term via vaginal delivery, weighing 2600 grams. She has been followed for developmental delay.

She can walk with support and speaks only single words. She has EEG abnormalities and a history of febrile seizures. There is no known parental consanguinity.

**CP_P43.1:** A male patient was born at term via vaginal delivery, weighing 2850 grams and measuring 52 cm. He received phototherapy for neonatal hyperbilirubinemia.

Global developmental delay was noted. He speaks only two words. No causal variant was detected in CES reanalysis.

**CP_P44.1:** A male patient was born at term via vaginal delivery. No postnatal complications were reported. He is being followed with a preliminary diagnosis of cerebral palsy. There are no dysmorphic features, but hand joint laxity is present. There is no known parental consanguinity. No causal variant was detected in CES reanalysis.

**CP_P45.1:** A male patient was born to a 26-year-old mother (G1P1) at 41 weeks of gestation, weighing 4200 grams. Prenatal follow-up was regular and screening results were normal. There were no complications in the perinatal period, and no history of seizures.

Head control was achieved at 4–5 months, and unsupported sitting at around 14 months. He is unable to walk and has unclear speech. Spasticity is present in the lower extremities, and scoliosis was noted. No causal variant was detected in CES reanalysis. There is no known parental consanguinity or similar disease in the family.

**CP_P46.1:** A male patient was born at term via cesarean section, weighing 3105 grams and measuring 49 cm. No complications occurred during the prenatal or perinatal periods, and neonatal intensive care was not required.

Head control was achieved at 2.5 months. Crawling and walking have not been attained. He speaks in single words. From the fifth month onwards, developmental delay, bradykinesia, and hypertonicity were observed. He underwent tendon release surgery at the ankles. EEG revealed epileptiform activity, and he is currently receiving sodium valproate treatment. There is first-degree parental consanguinity, and no similar case in the family.

**CP_P47.1**: She was born at term (4350 g, 51 cm) by cesarean section. There was no postnatal complication, including jaundice. Neonatal-onset hypotonia and tonic-clonic seizures were reported. Head control was achieved at 5 months, sitting without support at 8 months, crawling at 10 months, and independent walking at 15 months. She speaks single words. Cranial MRI findings are present. Her 4-year-old brother has a diagnosis of epilepsy. There is no known consanguinity between the parents.

**CP_P48.1**: A 10-year-old male was born at term to a 38-year-old mother (G3P2). Motor delay became evident around 4–5 months of age. He achieved independent walking at 3.5 years and speech at 5 years. His first seizure occurred at the age of 2 and is currently controlled with anti-seizure medication. Cranial MRI revealed diffuse cerebral atrophy. He is followed for hypotonia. He is currently attending school as an inclusive student. Physical examination revealed pes planus. Dysmorphic features include hypotelorism, prominent forehead, wide/flared eyebrows, prominent upper incisors, and synophrys.

**CP_P49.1**: A 3-year-old male was born at term (3650 g) to a 34-year-old mother via vaginal delivery (G2P2). The neonatal period was uneventful. Head control was achieved at 3 months; he was able to sit after 8 months but still cannot sit independently. He has been walking with support since 1 year of age but has not yet achieved independent ambulation. His expressive vocabulary includes 15–25 words; he can identify colors and shapes. There is no history of seizures. Sleep pattern is regular. Physical examination revealed spasticity in all extremities. No overt dysmorphic features were noted. There is no known parental consanguinity.

**CP_P50.1**: A male patient was born at term (3950 g) to a 28-year-old mother (G3P2) via vaginal delivery. There were tonic seizures twice within the first 24 hours of life. He required neonatal intensive care due to frequent daily seizures, which are now controlled with anti-seizure medication. EEG continues to show epileptic activity. He is non-verbal but can understand emotional cues. Behavioral features include bruxism and hand-biting during anger or excitement. He has not yet achieved independent sitting or walking (currently 6.5 years old). He is fed puréed food due to lack of chewing ability. Brain MRI revealed a thin corpus callosum, bilateral symmetric periventricular white matter hyperintensities, mild ventriculomegaly, and sulcal enlargement. Physical examination revealed nystagmus, strabismus, myopia, and astigmatism. No facial dysmorphism was observed. Widespread spasticity and increased deep tendon reflexes were noted in both upper and lower extremities.

**CP_P51.1**: A 4-year-old male was born to a 30-year-old mother (G2P2) at term with a birth weight of 3020 g. Antenatal follow-up revealed increased nuchal translucency. Head control was achieved at 4–5 months, and he is still unable to walk. Hypotonia became more prominent at 6 months. He began speaking a few words around age 1. EMG showed a sensorimotor polyneuropathy characterized by chronic axonal loss in both motor and sensory fibers. Neurological examination revealed gross motor delay without cognitive impairment. Ocular findings included nystagmus and astigmatism. Physical findings included relative macrocephaly and pectus carinatum. No spasticity was noted. Multiple dental caries were observed. There is no known parental consanguinity.

**CP_P52.1**: A 9-year-old female was born extremely preterm at 5.5 months of gestation and was hospitalized for 8 months postnatally. She required home oxygen therapy until the age of 3. She presents with global developmental delay. Seizure history is present. Currently (9 years, 2 months), she has head control and can sit with support. She is non-verbal, recognizes her parents, but has limited awareness. Cranial MRI revealed pontocerebellar hypoplasia. WES was performed previously and reanalysis did not identify any causal variants. There is no known consanguinity between the parents and no similar history in the family.

**CP_P53.1:** A 3-year-old female patient was referred due to developmental delay. The pregnancy was regularly monitored, and prenatal development was reported to be normal. Hypotonia was noted during infancy, and developmental delay became evident around 4–5 months of age. Head control was achieved at 6 months; however, the patient has never attained sitting, walking, or speech milestones. She demonstrates visual tracking and social smiling. Muscle tone is increased in all extremities with axial hypotonia. Deep tendon reflexes are brisk. No organomegaly was noted, but genital hypoplasia is present. There is no history of epileptic seizures. WES was previously performed due to unexplained cerebral palsy, but no diagnostic variant was identified. The patient was referred for reanalysis. The parents are non-consanguineous, and there is no family history of similarly affected individuals.

**CP_P54.1:** A 4-year-old female patient was born at term (40 weeks) via spontaneous vaginal delivery, with a birth weight of 3060 g. The first seizure occurred at 31 days of age and was described as tonic. She was hospitalized with a preliminary diagnosis of meningitis/encephalitis, but no infectious agent was identified. At 7 months of age, she was noted to have microcephaly (head circumference < -3 SD), hypotonia, absent supported sitting, and limited social smiling. Cranial MRI revealed cerebellar atrophy, reduced cerebellar volume, and ventriculomegaly. EEG abnormalities were also present. WES and mitochondrial genome analysis were performed but yielded negative results. At 2 years of age, her growth parameters remained below age expectations. She was alert and responsive but lacked age-appropriate reactions. Visual tracking was preserved. Neurological examination revealed marked hypotonia with distal hypertonia and dystonia. Ophthalmologic and audiologic evaluations were normal. The parents are non-consanguineous, and there is no family history of similar conditions. WES reanalysis did not identify any causative variant.

**CP_P55.1 & CP_P55.2:** This family has included two affected siblings born to non-consanguineous parents. The 7-year-old male patient began experiencing seizures at 5 months of age and has been on anti-seizure treatment since. He can sit independently but is unable to walk or speak. EEG was abnormal, while cranial MRI was unremarkable. Downward gaze abnormalities were noted. The 2-year-old female sibling, presented with similar concerns. At 2 months, she achieved head control and visual tracking. The family reported possible seizure-like episodes and downward gaze abnormalities, similar to her brother. EEG was also abnormal. The family has previously lost another daughter with similar symptoms. No causative variants were identified through WES reanalysis.

**CP_P56.1:** The 6-year-old male patient was born from a twin pregnancy via IVF, to a 34-year-old mother, at 34 weeks of gestation by cesarean section. His birth weight was 2400 g, and he was monitored in an incubator for 3 days. His twin sibling, who experienced a more severe hypoxic course and required neonatal intensive care. Motor delay in the patient became evident around 6 months of age. He achieved supported sitting by age 2, and ambulation was delayed until age 5. Due to significant spasticity, selective dorsal rhizotomy was performed, which improved muscle stiffness. He now walks with a walker. Over the past year, he has started speaking in sentences. He attends kindergarten with no reported cognitive or learning difficulties. No history of seizures or comorbid conditions was noted.
The mother had a childhood history of toe-walking and was diagnosed with cerebral palsy at age 12. On neurological exam, she shows bilateral lower limb spasticity, hyperreflexia, pes cavus, and a mild scissoring gait. The maternal uncle was diagnosed with late-onset ALS at age 55. Neurological examination of the proband revealed no dysmorphic features, but spasticity was present in all limbs. He could ambulate with a walker using a scissoring gait. Speech was somewhat unclear but affect was appropriate. Hearing and vision were normal.
No perinatal complication could explain the clinical findings. The unaffected twin sibling and the familial history raised suspicion for a genetic etiology. Therefore, trio-WES was performed, including the proband, mother, and twin sibling.

### CP_P57.1: This female patient was born to a 29-year-old mother at 38 weeks of gestation with a birth weight of 3150 g. The pregnancy and neonatal periods were unremarkable. Developmental milestones were mildly delayed: head control was achieved at 2 months, sitting at 7 months, first words at 9 months, and crawling after the age of 2 years. She is currently able to walk only with support or using a walker. She attends an inclusive education program and has begun learning to read and write. Seizures began at 5 months of age; however, EEG findings were normal. Brain MRI revealed volume loss in the cerebellar hemispheres and vermis; spinal MRI was unremarkable. Ophthalmological examination at age 1 revealed visual difficulties and prolonged epithelial latencies. Hearing was normal. On examination, she had a head circumference of 53 cm and weighed 25 kg. Dysmorphic features included narrow, upward-slanting palpebral fissures. She walked with knee flexion and in-toeing. Spasticity was present in the lower extremities. There is no known parental consanguinity.

### CP_P58.1: A 6-year-old female patient was born at term as the first child of a 24-year-old mother with a birth weight of 2500 g. Prenatal macrocephaly was noted. Due to respiratory distress, she required neonatal intensive care. Seizures began at 3 months of age, and she is currently on triple anti-seizure therapy with stable control. She can sit with support but has never crawled or walked. She has no verbal output and minimal responsive smiling. She has a good appetite but poor chewing ability. She attends special education with limited benefit. Constipation is a frequent issue. Ophthalmological exam was normal aside from mild strabismus. Cranial MRI showed corpus callosum hypogenesis, hydrocephalus, and colpocephaly. Hearing was normal.

### Dysmorphic features include brachycephaly, narrow forehead, mild proptosis, retrognathia, and a generally dysmorphic appearance. Spasticity was present in all extremities. Previous clinical exome analysis identified a **PAH c.1066T>C** variant, but metabolic workup did not support phenylketonuria. A sibling has autism but has not undergone genetic testing.

### CP_P59.1: This male patient was born at 35 weeks of gestation to a 33-year-old mother and required 23 days of incubator care due to feeding difficulties. Delayed head control and hypotonia were noted by 2 months of age. He has recently begun to sit with support and displays visual engagement and smiling.Ophthalmologic examination was normal. Metabolic tests were within normal limits. Cranial MRI, EEG, and exome sequencing are pending. He exhibits spasticity in all extremities (more pronounced in the lower limbs), a round face, and poor head control. A male sibling, now deceased, was born at 33 weeks after anhydramnios. He showed developmental delay and hypotonia from 6 months, and seizures began at age 3. MRI showed frontotemporal CSF prominence and cortical atrophy. He had dystonia, increased tone, hypoactive reflexes, extensor plantar responses, and hammer toes. Observed HPO terms in the current patient: round face, poor head control, global developmental delay, hypotonia, and spasticity.

### CP_P60.1: This female patient was born at term via spontaneous delivery, weighing 3600 g. She did not require neonatal intensive care admission. The parents are non-consanguineous, and no similarly affected individuals are reported in the family. Since 6 months of age, she has been followed for neuromotor delay, hypotonia, and microcephaly (−2 SD). Sitting was achieved after 1 year, and first words appeared around 1 year. Examination showed increased tone in the lower limbs, rocker bottom foot deformity, and global developmental delay. At 3 years, she experienced afebrile generalized tonic-clonic seizures. MRI at 10 months revealed mild ventriculomegaly and T2-FLAIR hyperintensities in the bilateral parieto-occipital periventricular regions. Chromosomal microarray and trio-WES analyses were not detected any causative variant.

### CP_P61.1: This 9-year-old male patient has a history of epilepsy, motor retardation, and spastic paraplegia. He was born at term, and not required neonatal intensive care admission. The parents are non-consanguineous. A sibling also has epilepsy. He achieved sitting at 1.5 years and walking with support after age 2. He has been wheelchair-bound for the past 2 years. Seizures began at 3 years of age. Initial metabolic screening was normal. Cranial MRI (2018) showed delayed myelination with focal white matter hyperintensities in the bilateral parietal deep white matter. Spinal MRI was normal. Genetic studies including chromosomal microarray and hereditary spastic paraplegia gene panel were non-diagnostic.

### CP_P62.1: An 18-year-old male patient with epilepsy and global developmental delay was born at 41 weeks via cesarean section, weighing 4300 g. He did not require neonatal intensive care admission. The parents are first cousins. A maternal aunt also has epilepsy. Seizures began at age 9. Walking started at 7 years but regressed by age 16. He has never developed speech. Neurological exam revealed increased tone, hyperreflexia, spastic tetraparesis, tremor, and stereotypic hand movements. EEG showed epileptiform discharges. Metabolic screening and prior genetic tests (karyotype, epilepsy panel) were normal.

### CP_P63.1: An 18-year-old female patient with moderate intellectual disability and spastic quadriparesis was born at term via cesarean section after a difficult delivery (birth weight 4800 g) at term. She required neonatal intensive care admission due to perinatal hypoxia. She began walking and talking around age 5–6. No history of seizures. MRI has not been performed. She walks with a scissoring gait. Physical exam showed spasticity in all limbs, joint laxity in fingers, pes cavus, and articulation difficulties. Her younger brother (currently 30) also has spastic quadriparesis and speech delay. He was hospitalized at 13 days for poor sucking. Developmental delays and instability in walking were noted. He has seizures and is followed with a diagnosis of spastic quadriparesis. No MRI was performed. Trio-WES analysis was not detected any causative variant.

CP_P64.1: This 10-year-old male patient began having seizures at 13 days of age. Developmental delay became evident by 7–8 months. He sat independently at 2.5 years but never walked or developed expressive language. He is currently non-verbal and wheelchair-bound. There are signs of neurological regression with global motor and cognitive decline. Cranial MRI and MR spectroscopy were reported as normal. EEG in 2021 showed generalized epileptiform discharges. Genetic testing including chromosomal microarray, epilepsy gene panel, Angelman/Prader-Willi syndrome testing, and **SLC2A1** screening were all normal.


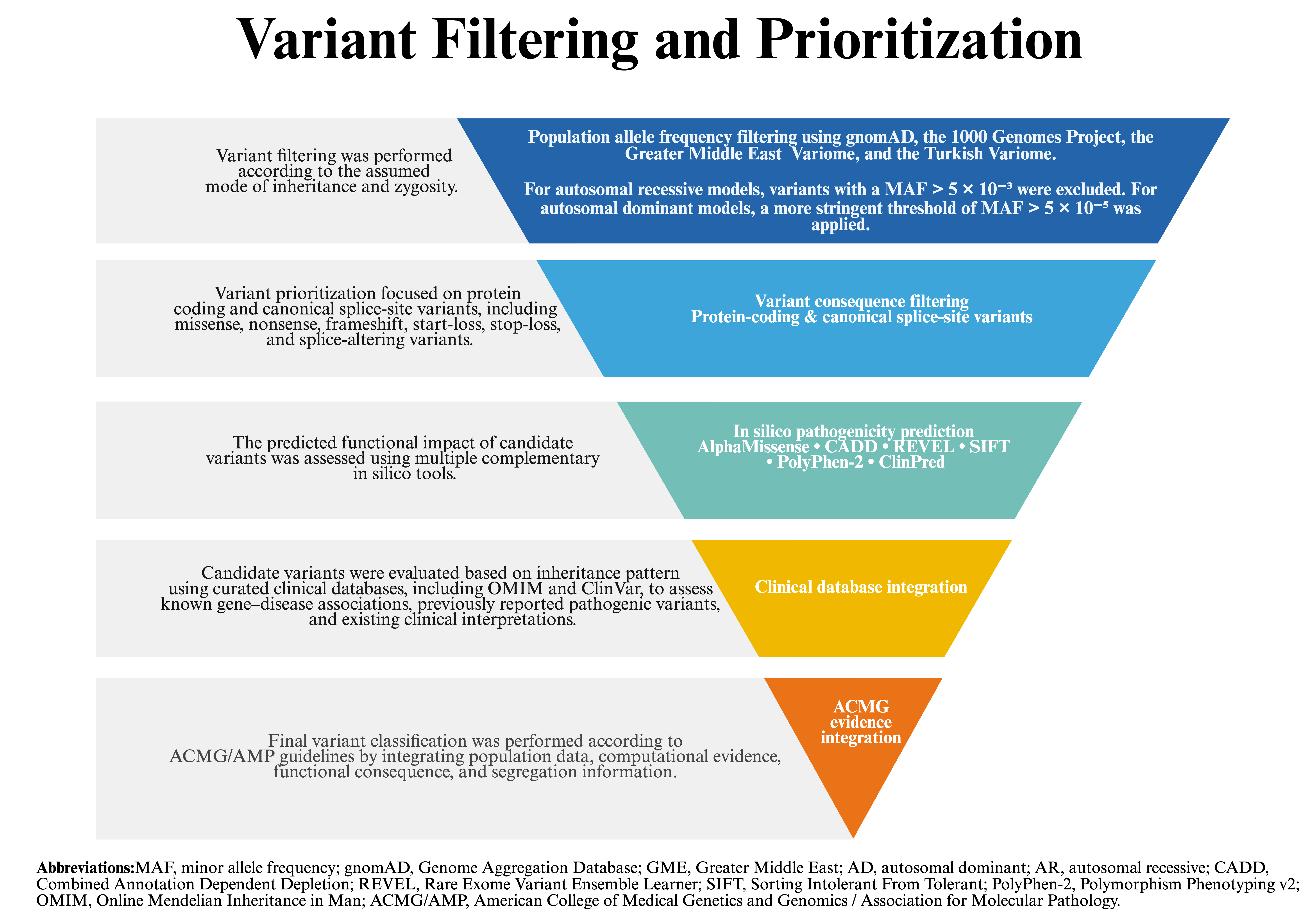


***Supplementary Figure 1. Variant prioritization and interpretation workflow***
Variants were prioritized through sequential filtering based on population allele frequency thresholds applied according to zygosity and inheritance model, variant consequence, integrative in silico pathogenicity prediction, clinical database review, and ACMG/AMP-based clinical interpretation to identify candidate disease-causing variants.


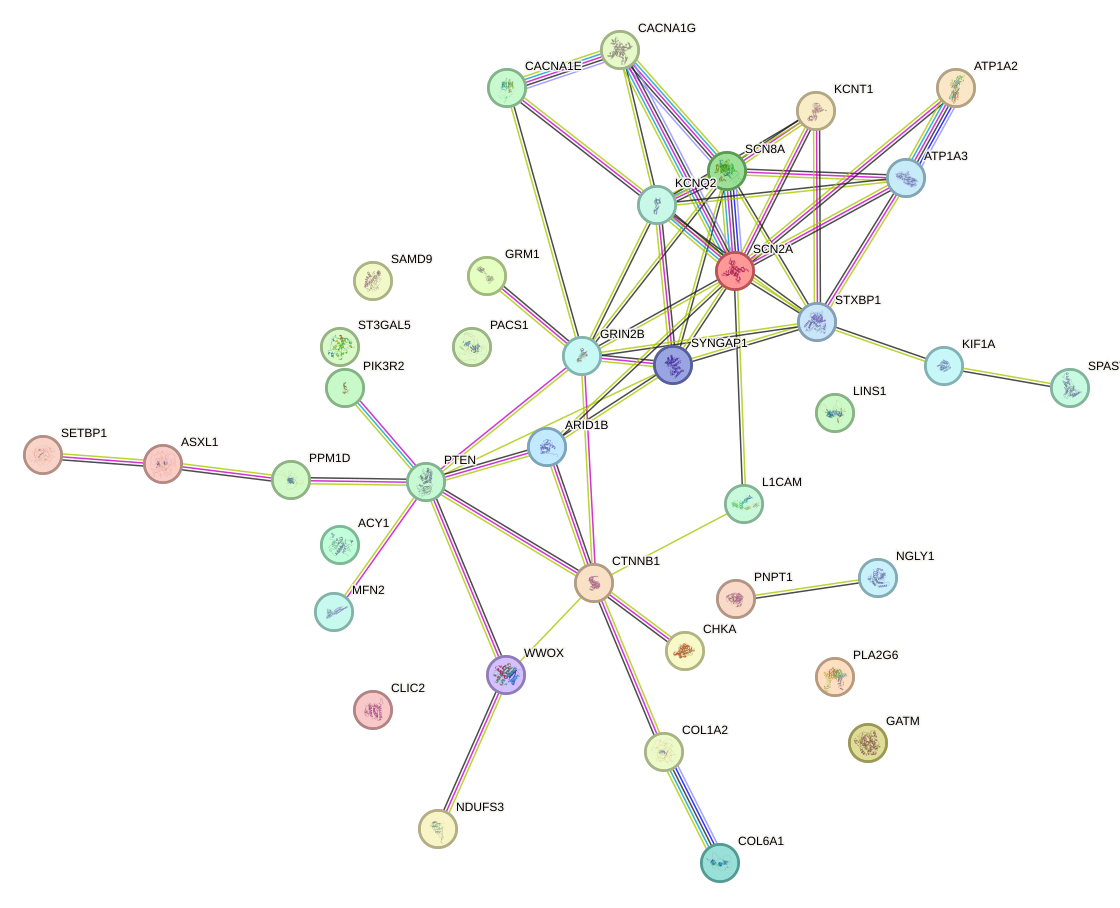


***Supplementary Figure 2. Protein–protein interaction (PPI) network of genes prioritized through variant interpretation in the Turkish CP cohort. The network was generated using STRING v12.0. Nodes represent proteins encoded by the prioritized genes, and edges indicate known or predicted functional associations. The network contains 38 nodes and 56 edges, significantly more than the 18 edges expected by chance (PPI enrichment p-value = 9.08 × 10⁻¹³), indicating a non-random, functionally coherent gene set. Edge colors represent evidence sources, including experimental data (pink), curated databases (blue), co-expression (black), and co-occurrence or text mining (green/yellow).***


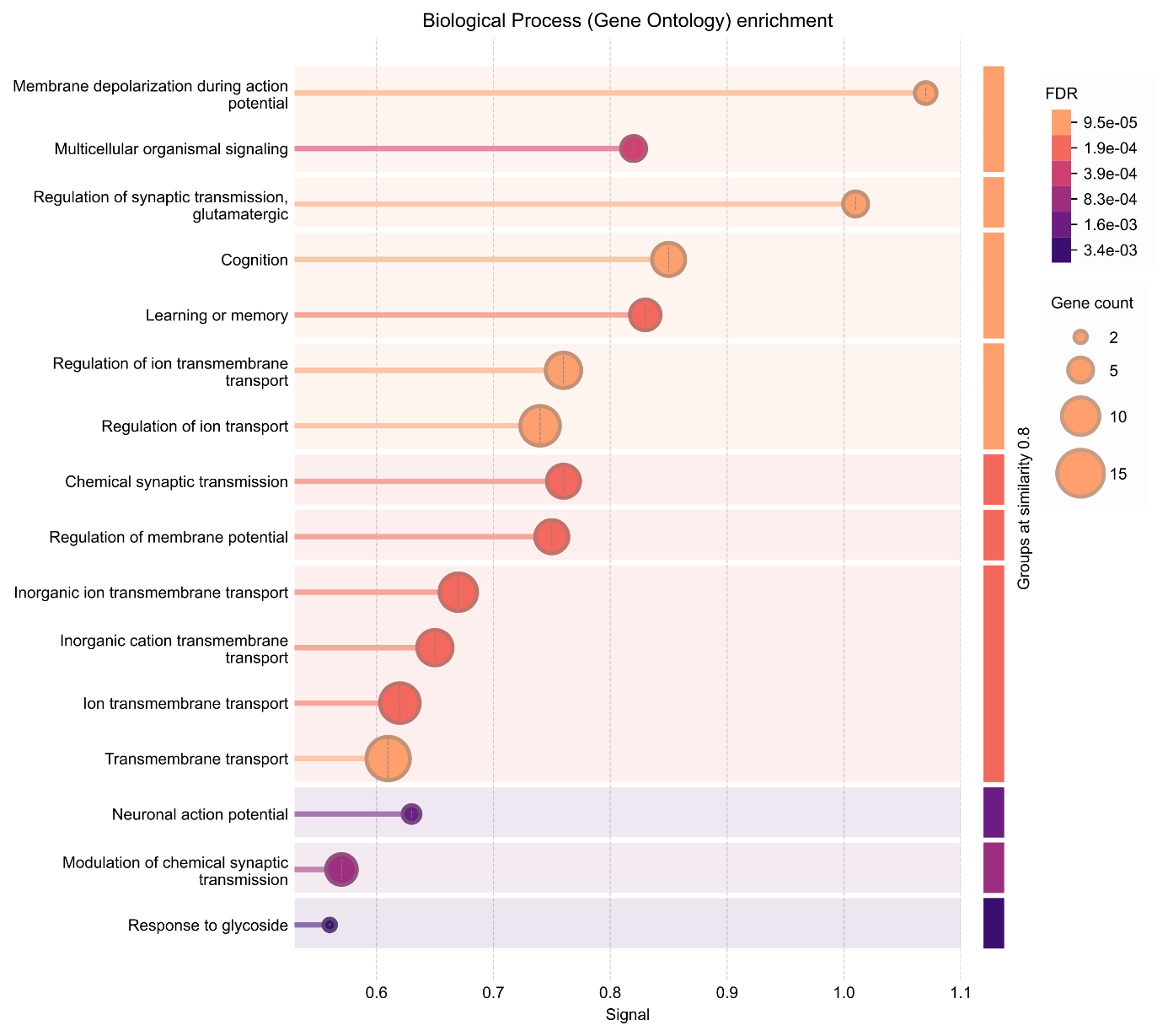


***Supplementary Figure 3. Highest ranking molecular function enrichment GO terms for biological process, sorted by signal.***


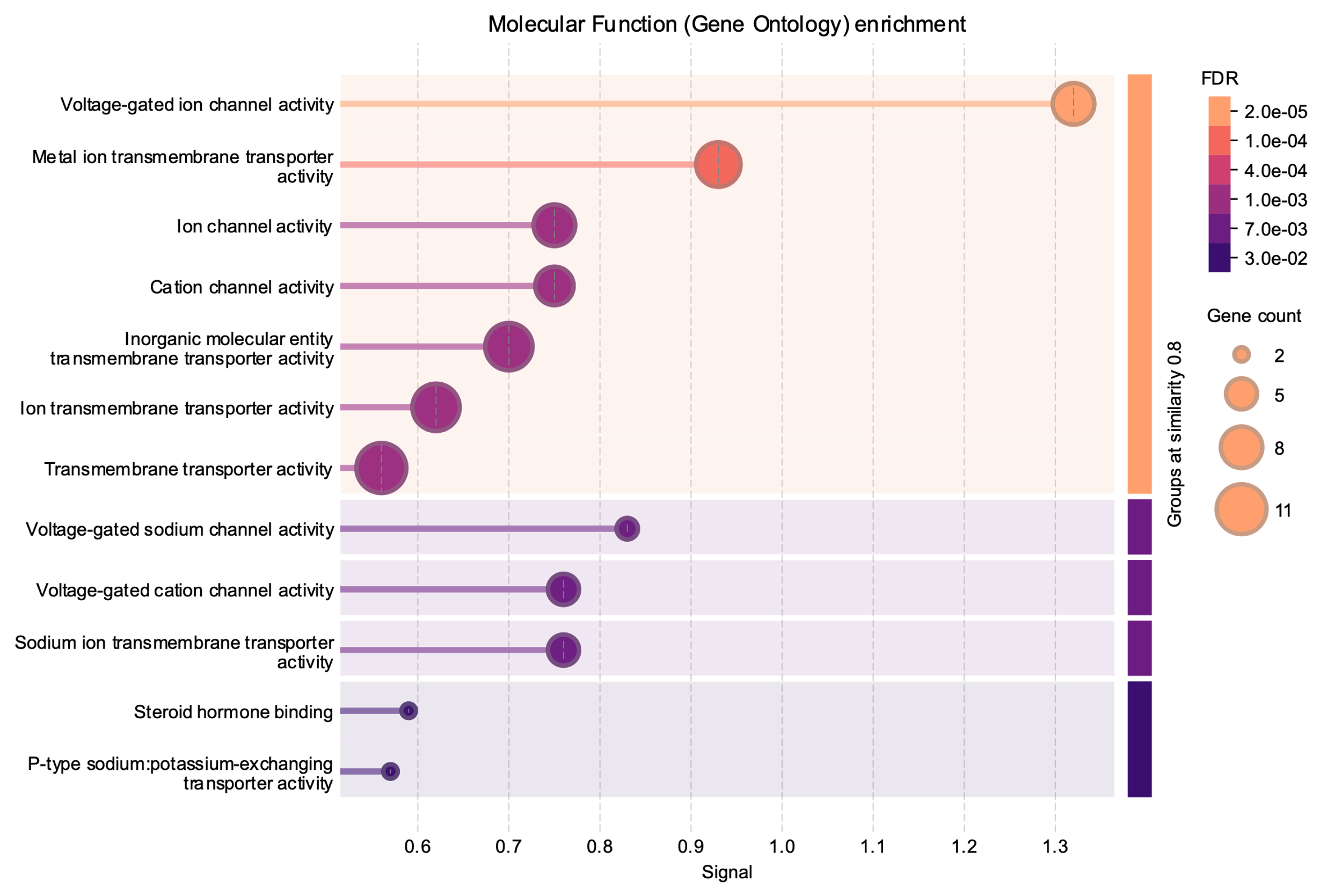


***Supplementary Figure 4. Highest ranking molecular function enrichment GO terms for biological process, sorted by signal.***

Molecular function analysis emphasized voltage-gated ion channel activity, metal ion transmembrane transporter activity, and steroid hormone binding, highlighting the contribution of ion channel dynamics and membrane-associated signaling.

***Supplementary Table 3. Highest ranking Biological process enrichment GO terms for biological process, sorted by signal.***

| #term ID | term description | observed gene count | background gene count | strength | signal | false discovery rate | matching proteins in the network |
| --- | --- | --- | --- | --- | --- | --- | --- |
| GO:0086010 | Membrane depolarization during action potential | 4 | 30 | 1.84 | 1.07 | 0.0011 | SCN8A,CACNA1G,ATP1A2,SCN2A |
| GO:0051966 | Regulation of synaptic transmission, glutamatergic | 5 | 76 | 1.53 | 1.01 | 0.0011 | GRM1,PLA2G6,ATP1A2,STXBP1,GRIN2B |
| GO:0050890 | Cognition | 8 | 313 | 1.12 | 0.85 | 0.0011 | LINS1,PLA2G6,ATP1A2,PTEN,GATM,GRIN2B,SCN2A,SYNGAP1 |
| GO:0007611 | Learning or memory | 7 | 271 | 1.13 | 0.83 | 0.0014 | PLA2G6,ATP1A2,PTEN,GATM,GRIN2B,SCN2A,SYNGAP1 |
| GO:0035637 | Multicellular organismal signaling | 5 | 124 | 1.32 | 0.82 | 0.003 | SCN8A,CACNA1G,ATP1A2,ATP1A3,SCN2A |
| GO:0034765 | Regulation of ion transmembrane transport | 9 | 504 | 0.97 | 0.76 | 0.0011 | PLA2G6,SCN8A,CACNA1G,KCNQ2,ATP1A2,CACNA1E,CLIC2,GRIN2B,SCN2A |
| GO:0007268 | Chemical synaptic transmission | 8 | 409 | 1.01 | 0.76 | 0.0015 | GRM1,CACNA1G,KCNQ2,CACNA1E,PTEN,STXBP1,GRIN2B,CTNNB1 |
| GO:0042391 | Regulation of membrane potential | 8 | 428 | 0.99 | 0.75 | 0.0015 | GRM1,SCN8A,CACNA1G,ATP1A2,PTEN,ATP1A3,GRIN2B,SCN2A |
| GO:0043269 | Regulation of ion transport | 11 | 700 | 0.91 | 0.74 | 0.00095 | PLA2G6,SCN8A,CACNA1G,KCNQ2,ATP1A2,CACNA1E,CLIC2,STXBP1,GRIN2B,SCN2A,CTNNB1 |
| GO:0098660 | Inorganic ion transmembrane transport | 10 | 743 | 0.84 | 0.67 | 0.0015 | SCN8A,CACNA1G,KCNQ2,ATP1A2,CACNA1E,CLIC2,KCNT1,ATP1A3,GRIN2B,SCN2A |
| GO:0098662 | Inorganic cation transmembrane transport | 9 | 649 | 0.86 | 0.65 | 0.0024 | SCN8A,CACNA1G,KCNQ2,ATP1A2,CACNA1E,KCNT1,ATP1A3,GRIN2B,SCN2A |
| GO:0019228 | Neuronal action potential | 3 | 33 | 1.67 | 0.63 | 0.0191 | SCN8A,CACNA1G,SCN2A |
| GO:0034220 | Ion transmembrane transport | 11 | 973 | 0.77 | 0.62 | 0.0015 | GRM1,SCN8A,CACNA1G,KCNQ2,ATP1A2,CACNA1E,CLIC2,KCNT1,ATP1A3,GRIN2B,SCN2A |
| GO:0055085 | Transmembrane transport | 13 | 1294 | 0.72 | 0.61 | 0.0011 | NDUFS3,GRM1,SCN8A,CACNA1G,KCNQ2,ATP1A2,CACNA1E,CLIC2,KCNT1,PNPT1,ATP1A3,GRIN2B,SCN2A |
| GO:0050804 | Modulation of chemical synaptic transmission | 7 | 436 | 0.92 | 0.57 | 0.0096 | GRM1,PLA2G6,ATP1A2,PTEN,STXBP1,GRIN2B,SYNGAP1 |
| GO:1903416 | Response to glycoside | 2 | 6 | 2.24 | 0.56 | 0.0347 | ATP1A2,ATP1A3 |

***Supplementary Table 4. Highest ranking GO terms for molecular function, sorted by signal.***

| #term ID | term description | observed gene count | background gene count | strength | signal | false discovery rate | matching proteins in the network |
| --- | --- | --- | --- | --- | --- | --- | --- |
| GO:0005244 | Voltage-gated ion channel activity | 8 | 201 | 1.31 | 1.32 | 2.54E-05 | SCN8A,CACNA1G,KCNQ2,CACNA1E,CLIC2,KCNT1,GRIN2B,SCN2A |
| GO:0046873 | Metal ion transmembrane transporter activity | 9 | 432 | 1.03 | 0.93 | 0.00019 | SCN8A,CACNA1G,KCNQ2,ATP1A2,CACNA1E,KCNT1,ATP1A3,GRIN2B,SCN2A |
| GO:0015318 | Inorganic molecular entity transmembrane transporter activity | 10 | 735 | 0.85 | 0.7 | 0.00099 | SCN8A,CACNA1G,KCNQ2,ATP1A2,CACNA1E,CLIC2,KCNT1,ATP1A3,GRIN2B,SCN2A |
| GO:0005216 | Ion channel activity | 8 | 437 | 0.98 | 0.75 | 0.0014 | SCN8A,CACNA1G,KCNQ2,CACNA1E,CLIC2,KCNT1,GRIN2B,SCN2A |
| GO:0005261 | Cation channel activity | 7 | 337 | 1.03 | 0.75 | 0.002 | SCN8A,CACNA1G,KCNQ2,CACNA1E,KCNT1,GRIN2B,SCN2A |
| GO:0015075 | Ion transmembrane transporter activity | 10 | 851 | 0.78 | 0.62 | 0.002 | SCN8A,CACNA1G,KCNQ2,ATP1A2,CACNA1E,CLIC2,KCNT1,ATP1A3,GRIN2B,SCN2A |
| GO:0022857 | Transmembrane transporter activity | 11 | 1121 | 0.71 | 0.56 | 0.0023 | NDUFS3,SCN8A,CACNA1G,KCNQ2,ATP1A2,CACNA1E,CLIC2,KCNT1,ATP1A3,GRIN2B,SCN2A |
| GO:0015081 | Sodium ion transmembrane transporter activity | 5 | 153 | 1.23 | 0.76 | 0.0041 | SCN8A,CACNA1G,ATP1A2,ATP1A3,SCN2A |
| GO:0022843 | Voltage-gated cation channel activity | 5 | 151 | 1.23 | 0.76 | 0.0041 | CACNA1G,KCNQ2,CACNA1E,KCNT1,GRIN2B |
| GO:0005248 | Voltage-gated sodium channel activity | 3 | 24 | 1.81 | 0.83 | 0.0054 | SCN8A,CACNA1G,SCN2A |
| GO:1990239 | Steroid hormone binding | 2 | 6 | 2.24 | 0.59 | 0.0275 | ATP1A2,ATP1A3 |
| GO:0005391 | P-type sodium:potassium-exchanging transporter activity | 2 | 7 | 2.17 | 0.57 | 0.0318 | ATP1A2,ATP1A3 |


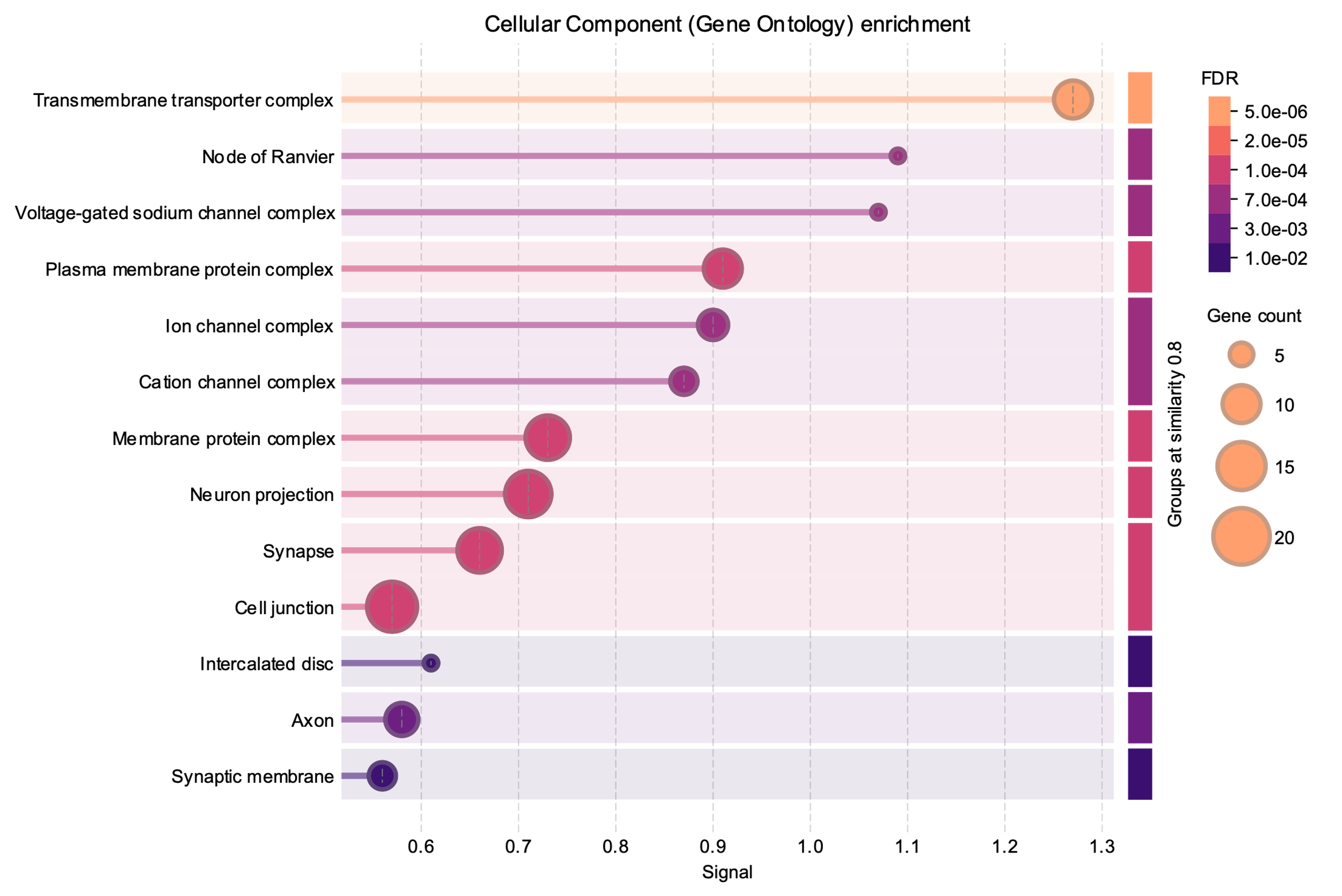


***Supplementary Figure 5. Cellular component enrichment revealed a focused localization of gene products to neuronal and membrane-associated structures. Key compartments included the transmembrane transporter complex, membrane and plasma membrane protein complexes, ion channel complexes, as well as structural features critical for neural conductivity such as the axon, node of Ranvier, and neuron projection.***

***Supplementary Table 5. Highest ranking GO terms for cellular component, sorted by signal.***

| #term ID | term description | observed gene count | background gene count | strength | signal | false discovery rate | matching proteins in the network |
| --- | --- | --- | --- | --- | --- | --- | --- |
| GO:1902495 | Transmembrane transporter complex | 10 | 384 | 1.13 | 1.27 | 5.27E-06 | NDUFS3,SCN8A,CACNA1G,KCNQ2,ATP1A2,CACNA1E,CLIC2,ATP1A3,GRIN2B,SCN2A |
| GO:0043005 | Neuron projection | 14 | 1391 | 0.72 | 0.71 | 9.22E-05 | GRM1,SPAST,SCN8A,CACNA1G,KCNQ2,ATP1A2,L1CAM,PTEN,STXBP1,KIF1A,ATP1A3,GRIN2B,SCN2A,SYNGAP1 |
| GO:0098797 | Plasma membrane protein complex | 10 | 589 | 0.94 | 0.91 | 9.22E-05 | GRM1,SCN8A,CACNA1G,KCNQ2,ATP1A2,CACNA1E,ATP1A3,GRIN2B,SCN2A,CTNNB1 |
| GO:0098796 | Membrane protein complex | 13 | 1218 | 0.74 | 0.73 | 0.0001 | PIK3R2,NDUFS3,GRM1,SCN8A,CACNA1G,KCNQ2,ATP1A2,CACNA1E,CLIC2,ATP1A3,GRIN2B,SCN2A,CTNNB1 |
| GO:0030054 | Cell junction | 16 | 2115 | 0.59 | 0.57 | 0.00024 | PIK3R2,GRM1,SCN8A,CACNA1G,KCNQ2,ATP1A2,CACNA1E,L1CAM,PTEN,STXBP1,KIF1A,ATP1A3,GRIN2B,SCN2A,CTNNB1,SYNGAP1 |
| GO:0045202 | Synapse | 13 | 1350 | 0.7 | 0.66 | 0.00024 | GRM1,CACNA1G,KCNQ2,ATP1A2,CACNA1E,PTEN,STXBP1,KIF1A,ATP1A3,GRIN2B,SCN2A,CTNNB1,SYNGAP1 |
| GO:0034702 | Ion channel complex | 7 | 300 | 1.08 | 0.9 | 0.00044 | SCN8A,CACNA1G,KCNQ2,CACNA1E,CLIC2,GRIN2B,SCN2A |
| GO:0120025 | Plasma membrane bounded cell projection | 16 | 2268 | 0.56 | 0.53 | 0.00044 | GRM1,SPAST,PLA2G6,SCN8A,CACNA1G,KCNQ2,ATP1A2,L1CAM,PTEN,STXBP1,KIF1A,ATP1A3,GRIN2B,SCN2A,CTNNB1,SYNGAP1 |
| GO:0034703 | Cation channel complex | 6 | 225 | 1.14 | 0.87 | 0.0009 | SCN8A,CACNA1G,KCNQ2,CACNA1E,GRIN2B,SCN2A |
| GO:0033268 | Node of Ranvier | 3 | 16 | 1.99 | 1.09 | 0.0011 | SCN8A,KCNQ2,SCN2A |
| GO:0001518 | Voltage-gated sodium channel complex | 3 | 17 | 1.96 | 1.07 | 0.0012 | SCN8A,CACNA1G,SCN2A |
| GO:0030424 | Axon | 8 | 651 | 0.8 | 0.58 | 0.0041 | SPAST,SCN8A,KCNQ2,L1CAM,STXBP1,KIF1A,ATP1A3,SCN2A |
| GO:0097060 | Synaptic membrane | 6 | 375 | 0.92 | 0.56 | 0.0104 | GRM1,STXBP1,ATP1A3,GRIN2B,SCN2A,CTNNB1 |
| GO:0014704 | Intercalated disc | 3 | 51 | 1.48 | 0.61 | 0.0184 | ATP1A2,SCN2A,CTNNB1 |
| GO:0098794 | Postsynapse | 7 | 621 | 0.77 | 0.45 | 0.0194 | GRM1,ATP1A2,STXBP1,ATP1A3,GRIN2B,CTNNB1,SYNGAP1 |
| GO:0042734 | Presynaptic membrane | 4 | 146 | 1.15 | 0.55 | 0.0208 | STXBP1,ATP1A3,SCN2A,CTNNB1 |
| GO:0005890 | Sodium:potassium-exchanging ATPase complex | 2 | 12 | 1.94 | 0.56 | 0.0316 | ATP1A2,ATP1A3 |
| GO:0032991 | Protein-containing complex | 21 | 5506 | 0.3 | 0.26 | 0.0344 | PIK3R2,NDUFS3,GRM1,COL1A2,SCN8A,CACNA1G,KCNQ2,ATP1A2,COL6A1,CACNA1E,CLIC2,STXBP1,ASXL1,PNPT1,KIF1A,ATP1A3,WWOX,GRIN2B,SCN2A,ARID1B,CTNNB1 |


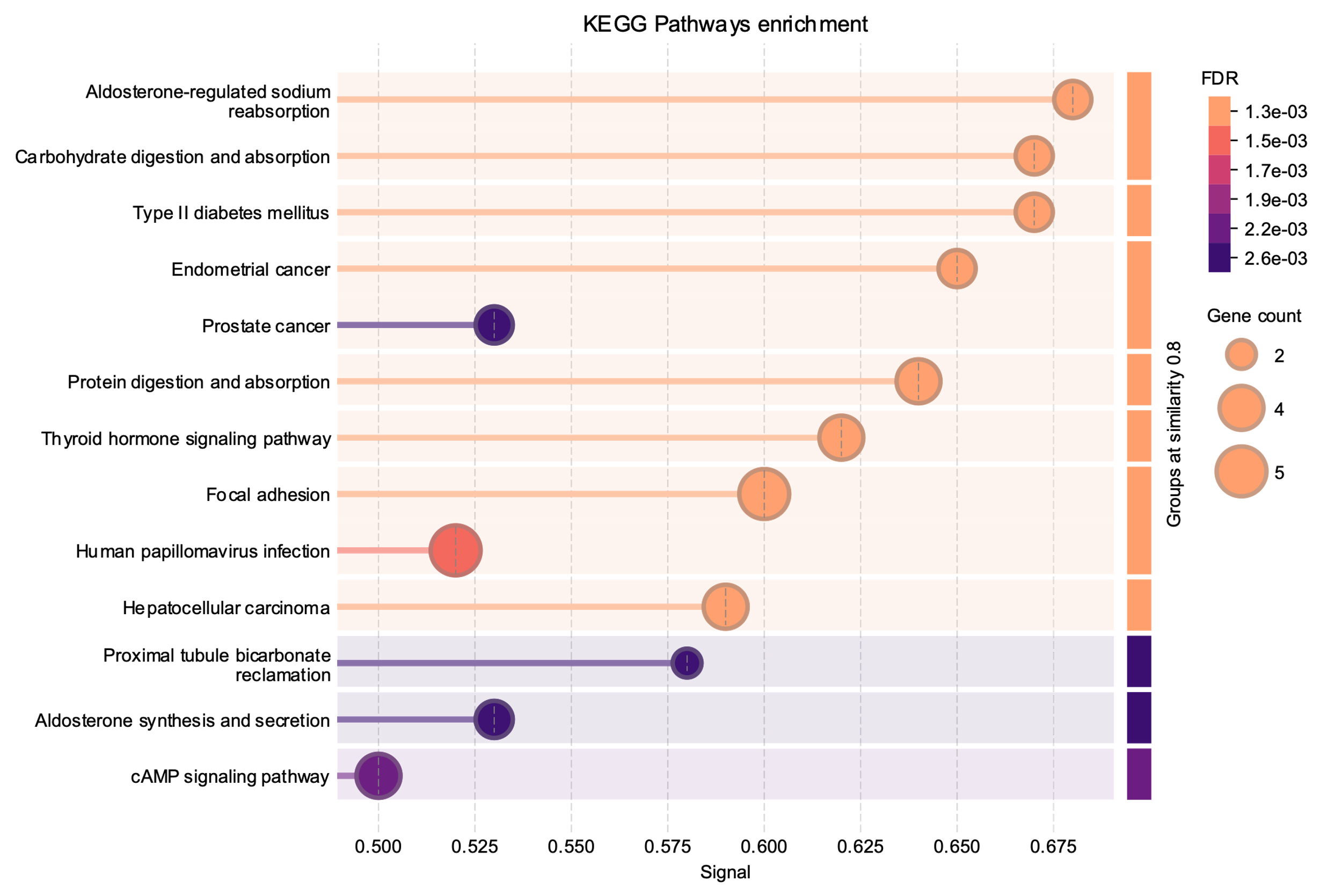


***Supplementary Figure 6. KEGG pathway analysis revealed associations with signaling and disease-relevant pathways such as the cAMP and Ras signaling pathways, thyroid hormone signaling, and aldosterone-regulated sodium reabsorption, as well as Type II diabetes, endometrial cancer, prostate cancer, and human papillomavirus infection.***

***Supplementary Table 6. Highest ranking KEGG pathway terms, sorted by signal.***

| #term ID | term description | observed gene count | background gene count | strength | signal | false discovery rate | matching proteins in your network (labels) |
| --- | --- | --- | --- | --- | --- | --- | --- |
| hsa04510 | Focal adhesion | 5 | 195 | 1.12 | 0.6 | 0.0131 | PIK3R2,COL1A2,COL6A1,PTEN,CTNNB1 |
| hsa04919 | Thyroid hormone signaling pathway | 4 | 120 | 1.24 | 0.62 | 0.0131 | PIK3R2,ATP1A2,ATP1A3,CTNNB1 |
| hsa04930 | Type II diabetes mellitus | 3 | 45 | 1.54 | 0.67 | 0.0131 | PIK3R2,CACNA1G,CACNA1E |
| hsa04960 | Aldosterone-regulated sodium reabsorption | 3 | 37 | 1.62 | 0.68 | 0.0131 | PIK3R2,ATP1A2,ATP1A3 |
| hsa04973 | Carbohydrate digestion and absorption | 3 | 44 | 1.55 | 0.67 | 0.0131 | PIK3R2,ATP1A2,ATP1A3 |
| hsa04974 | Protein digestion and absorption | 4 | 100 | 1.32 | 0.64 | 0.0131 | COL1A2,ATP1A2,COL6A1,ATP1A3 |
| hsa05213 | Endometrial cancer | 3 | 58 | 1.43 | 0.65 | 0.0131 | PIK3R2,PTEN,CTNNB1 |
| hsa05225 | Hepatocellular carcinoma | 4 | 161 | 1.11 | 0.59 | 0.0131 | PIK3R2,PTEN,ARID1B,CTNNB1 |
| hsa05165 | Human papillomavirus infection | 5 | 324 | 0.9 | 0.52 | 0.0149 | PIK3R2,COL1A2,COL6A1,PTEN,CTNNB1 |
| hsa04024 | cAMP signaling pathway | 4 | 207 | 1 | 0.5 | 0.0237 | PIK3R2,ATP1A2,ATP1A3,GRIN2B |
| hsa04014 | Ras signaling pathway | 4 | 225 | 0.96 | 0.48 | 0.0262 | PIK3R2,PLA2G6,GRIN2B,SYNGAP1 |
| hsa04925 | Aldosterone synthesis and secretion | 3 | 94 | 1.22 | 0.53 | 0.0262 | CACNA1G,ATP1A2,ATP1A3 |
| hsa04964 | Proximal tubule bicarbonate reclamation | 2 | 22 | 1.67 | 0.58 | 0.0262 | ATP1A2,ATP1A3 |
| hsa05215 | Prostate cancer | 3 | 97 | 1.21 | 0.53 | 0.0262 | PIK3R2,PTEN,CTNNB1 |
| hsa04068 | FoxO signaling pathway | 3 | 126 | 1.09 | 0.45 | 0.0436 | PIK3R2,GRM1,PTEN |
| hsa05017 | Spinocerebellar ataxia | 3 | 135 | 1.06 | 0.43 | 0.0495 | PIK3R2,GRM1,GRIN2B |


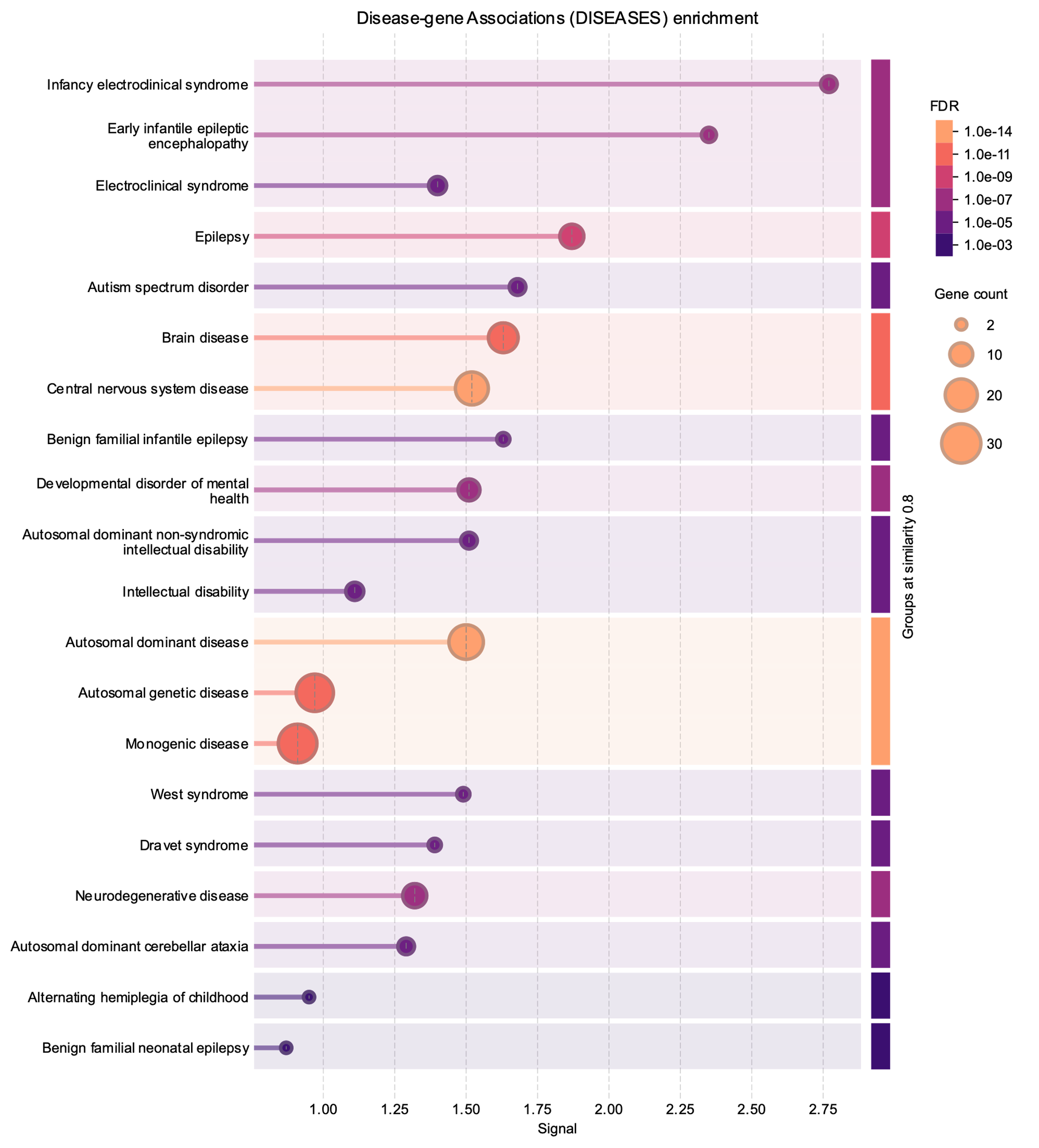


***Supplementary Figure 7. DISEASES enrichment revealed strong associations with a spectrum of neurological, genetic, and neurodevelopmental disorders, including autosomal dominant disease, monogenic disease, epilepsy, autism spectrum disorder, intellectual disability, West syndrome, Dravet syndrome, Lennox-Gastaut syndrome, and early infantile epileptic encephalopathy.***

***Supplementary Table 7. Highest ranking DISEASES terms, sorted by signal.***

| #term ID | term description | observed gene count | background gene count | strength | signal | false discovery rate | matching proteins in the network |
| --- | --- | --- | --- | --- | --- | --- | --- |
| DOID:0050736 | Autosomal dominant disease | 23 | 1386 | 0.93 | 1.5 | 9.09E-14 | GRM1,COL1A2,PACS1,SPAST,PLA2G6,SCN8A,CACNA1G,KCNQ2,ATP1A2,COL6A1,KCNT1,PTEN,STXBP1,ASXL1,PNPT1,MFN2,KIF1A,ATP1A3,WWOX,SCN2A,ARID1B,SYNGAP1,SETBP1 |
| DOID:331 | Central nervous system disease | 21 | 1199 | 0.96 | 1.52 | 8.34E-13 | GRM1,SPAST,PLA2G6,SCN8A,CACNA1G,KCNQ2,ATP1A2,CACNA1E,L1CAM,KCNT1,PTEN,STXBP1,PNPT1,MFN2,KIF1A,ATP1A3,WWOX,GRIN2B,SCN2A,ST3GAL5,CTNNB1 |
| DOID:0050177 | Monogenic disease | 29 | 3266 | 0.66 | 0.91 | 1.33E-12 | NGLY1,GRM1,COL1A2,PACS1,SPAST,PLA2G6,SCN8A,CACNA1G,KCNQ2,ATP1A2,COL6A1,CLIC2,L1CAM,KCNT1,PTEN,STXBP1,ASXL1,GATM,PNPT1,MFN2,KIF1A,ATP1A3,WWOX,SCN2A,ARID1B,ST3GAL5,CTNNB1,SYNGAP1,SETBP1 |
| DOID:0050739 | Autosomal genetic disease | 27 | 2802 | 0.7 | 0.97 | 3.63E-12 | NGLY1,GRM1,COL1A2,PACS1,SPAST,PLA2G6,SCN8A,CACNA1G,KCNQ2,ATP1A2,COL6A1,KCNT1,PTEN,STXBP1,ASXL1,GATM,PNPT1,MFN2,KIF1A,ATP1A3,WWOX,SCN2A,ARID1B,ST3GAL5,CTNNB1,SYNGAP1,SETBP1 |
| DOID:630 | Genetic disease | 30 | 3778 | 0.61 | 0.83 | 3.63E-12 | NDUFS3,NGLY1,GRM1,COL1A2,PACS1,SPAST,PLA2G6,SCN8A,CACNA1G,KCNQ2,ATP1A2,COL6A1,CLIC2,L1CAM,KCNT1,PTEN,STXBP1,ASXL1,GATM,PNPT1,MFN2,KIF1A,ATP1A3,WWOX,SCN2A,ARID1B,ST3GAL5,CTNNB1,SYNGAP1,SETBP1 |
| DOID:936 | Brain disease | 17 | 806 | 1.04 | 1.63 | 2.87E-11 | GRM1,PLA2G6,SCN8A,CACNA1G,KCNQ2,ATP1A2,CACNA1E,L1CAM,KCNT1,STXBP1,PNPT1,KIF1A,ATP1A3,WWOX,GRIN2B,SCN2A,ST3GAL5 |
| DOID:4 | Disease | 34 | 6291 | 0.45 | 0.59 | 1.63E-10 | NDUFS3,NGLY1,GRM1,COL1A2,PPM1D,PACS1,SPAST,PLA2G6,SCN8A,CACNA1G,KCNQ2,ATP1A2,COL6A1,CACNA1E,CLIC2,L1CAM,KCNT1,PTEN,STXBP1,ASXL1,SAMD9,GATM,PNPT1,MFN2,KIF1A,ATP1A3,WWOX,GRIN2B,SCN2A,ARID1B,ST3GAL5,CTNNB1,SYNGAP1,SETBP1 |
| DOID:1826 | Epilepsy | 11 | 306 | 1.27 | 1.87 | 7.25E-09 | SCN8A,KCNQ2,ATP1A2,CACNA1E,KCNT1,STXBP1,ATP1A3,WWOX,GRIN2B,SCN2A,ST3GAL5 |
| DOID:0050703 | Infancy electroclinical syndrome | 5 | 10 | 2.41 | 2.77 | 3.04E-08 | SCN8A,KCNQ2,KCNT1,STXBP1,SCN2A |
| DOID:0050709 | Early infantile epileptic encephalopathy | 4 | 5 | 2.62 | 2.35 | 5.66E-07 | SCN8A,KCNQ2,STXBP1,SCN2A |
| DOID:1289 | Neurodegenerative disease | 11 | 481 | 1.07 | 1.32 | 5.66E-07 | GRM1,SPAST,PLA2G6,CACNA1G,ATP1A2,L1CAM,PNPT1,MFN2,KIF1A,ATP1A3,WWOX |
| DOID:7 | Disease of anatomical entity | 27 | 4798 | 0.46 | 0.56 | 6.48E-07 | GRM1,COL1A2,PPM1D,SPAST,PLA2G6,SCN8A,CACNA1G,KCNQ2,ATP1A2,COL6A1,CACNA1E,L1CAM,KCNT1,PTEN,STXBP1,ASXL1,PNPT1,MFN2,KIF1A,ATP1A3,WWOX,GRIN2B,SCN2A,ST3GAL5,CTNNB1,SYNGAP1,SETBP1 |
| DOID:0060037 | Developmental disorder of mental health | 9 | 273 | 1.23 | 1.51 | 8.13E-07 | PACS1,CLIC2,PTEN,KIF1A,GRIN2B,SCN2A,ARID1B,SYNGAP1,SETBP1 |
| DOID:0060041 | Autism spectrum disorder | 5 | 45 | 1.76 | 1.68 | 1.15E-05 | PTEN,GRIN2B,SCN2A,ARID1B,SYNGAP1 |
| DOID:0050701 | Electroclinical syndrome | 6 | 114 | 1.44 | 1.4 | 2.68E-05 | SCN8A,KCNQ2,KCNT1,STXBP1,WWOX,SCN2A |
| DOID:0060307 | Autosomal dominant non-syndromic intellectual disability | 5 | 57 | 1.66 | 1.51 | 2.91E-05 | PACS1,KIF1A,ARID1B,SYNGAP1,SETBP1 |
| DOID:0060169 | Benign familial infantile epilepsy | 3 | 4 | 2.59 | 1.63 | 5.02E-05 | SCN8A,KCNQ2,SCN2A |
| DOID:0050562 | West syndrome | 3 | 6 | 2.41 | 1.49 | 0.00011 | KCNT1,STXBP1,SCN2A |
| DOID:1441 | Autosomal dominant cerebellar ataxia | 5 | 78 | 1.52 | 1.29 | 0.00011 | GRM1,CACNA1G,ATP1A2,PNPT1,WWOX |
| DOID:1059 | Intellectual disability | 6 | 166 | 1.27 | 1.11 | 0.00017 | PACS1,CLIC2,KIF1A,ARID1B,SYNGAP1,SETBP1 |
| DOID:0080422 | Dravet syndrome | 3 | 8 | 2.29 | 1.39 | 0.0002 | SCN8A,STXBP1,SCN2A |
| DOID:0050635 | Alternating hemiplegia of childhood | 2 | 2 | 2.71 | 0.95 | 0.0033 | ATP1A2,ATP1A3 |
| DOID:14777 | Benign familial neonatal epilepsy | 2 | 3 | 2.54 | 0.87 | 0.0053 | KCNQ2,SCN2A |
| DOID:0060246 | MASA syndrome | 2 | 4 | 2.41 | 0.8 | 0.0077 | SPAST,L1CAM |
| DOID:0050561 | Lennox-Gastaut syndrome | 2 | 8 | 2.11 | 0.63 | 0.0217 | SCN8A,STXBP1 |
| DOID:182 | Calcinosis | 2 | 10 | 2.02 | 0.57 | 0.0309 | COL6A1,SAMD9 |
| DOID:1036 | Chronic leukemia | 2 | 11 | 1.97 | 0.55 | 0.0355 | ASXL1,SETBP1 |
